# Discovery of protein acetylation patterns by deconvolution of peptide isomer mass spectra

**DOI:** 10.1038/ncomms9648

**Published:** 2015-10-15

**Authors:** Nebiyu Abshiru, Olivier Caron-Lizotte, Roshan Elizabeth Rajan, Adil Jamai, Christelle Pomies, Alain Verreault, Pierre Thibault

**Affiliations:** 1 Department of Chemistry, Université de Montréal, PO Box 6128, Station centre-ville, Montréal, Québec, Canada H3C 3J7; 2 Institute for Research in Immunology and Cancer, Université de Montréal, C.P. 6128, Succursale centre-ville, Montréal, Québec, Canada H3C 3J7; 3 Molecular Biology Programme, Université de Montréal, PO Box 6128, Station centre-ville, Montréal, Québec, Canada H3C 3J7

## Abstract

Protein post-translational modifications (PTMs) play important roles in the control of various biological processes including protein–protein interactions, epigenetics and cell cycle regulation. Mass spectrometry-based proteomics approaches enable comprehensive identification and quantitation of numerous types of PTMs. However, the analysis of PTMs is complicated by the presence of indistinguishable co-eluting isomeric peptides that result in composite spectra with overlapping features that prevent the identification of individual components. In this study, we present Iso-PeptidAce, a novel software tool that enables deconvolution of composite MS/MS spectra of isomeric peptides based on features associated with their characteristic fragment ion patterns. We benchmark Iso-PeptidAce using dilution series prepared from mixtures of known amounts of synthetic acetylated isomers. We also demonstrate its applicability to different biological problems such as the identification of site-specific acetylation patterns in histones bound to chromatin assembly factor-1 and profiling of histone acetylation in cells treated with different classes of HDAC inhibitors.

## Abstract (web summary)

Deciphering patterns of histone modifications that modulate chromatin structure and function is important, but remains challenging. Here the authors describe a method to uncover patterns of site-specific histone acetylation by deconvolution of overlapping peptide isomer mass spectra.

## Graphical abstract

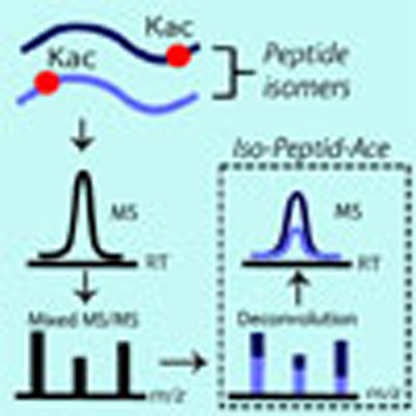

Histone post-translational modifications (PTMs) control access to genetic information and, consequently, participate in several important cellular processes such as DNA repair and replication, nucleosome assembly and transcriptional regulation^1^,^2^. It is now clear that many chromatin-modifying enzymes and enzymes that act on non-histone proteins, recognize ‘combinatorial patterns of PTMs', rather than single protein modifications. For instance, ATP-dependent chromatin remodelers often recognize histones that contain more than one acetylated lysine residue that are located in close proximity of each other^3^,^4^. Although valuable to infer the functions of PTMs, accurately determining patterns and stoichiometries of PTMs located in isomeric and isobaric peptides remains extremely difficult to achieve even by mass spectrometry (MS). This is because isomeric peptides, which are identical except for the position of the PTMs along the peptide chain, often co-elute during liquid chromatography and generate composite tandem mass spectra (MS/MS) containing indistinguishable fragment ions derived from two or more peptide isomers.

A few MS/MS methods were previously developed to quantify co-eluting isobaric histone peptides. Smith *et al.*^5^ were first to use a fragment ion-based approach to determine the fraction of histone H4 molecules acetylated at specific residues based on the normalized intensity ratio of peptide fragment ions containing either protiated or deuterated acetyl groups to distinguish lysine residues that were, respectively, acetylated or free *in vivo*. However, this approach was limited by the lack of automated data analysis software and the difficulty to deconvolute co-eluting isomers. Recently, Feller *et al.*^6^ described a similar approach in which second generation fragment ions (known as MS^3^) were necessary to obtain diagnostic fragment ions that can be used to estimate the abundance of each isomer by solving a set of linear equations. A limitation of this approach is that singly charged MS^2^ fragments, and particularly those with a nonmobile proton, fragment poorly and generate MS^3^ spectra that are impossible to assign to specific isomers^7^,^8^. Sidoli *et al.*^9^ designed a software known as isoScale that can be used to determine the relative abundance of isobaric peptides, based on intensity ratios of fragment ions unique to each isomer. isoScale relies on search engine outputs (for example, Mascot.csv result files) to select isomer-specific fragments used in this analysis. The algorithm EpiProfile recently described by Yuan *et al.*^10^ quantifies isomeric histone peptides by solving a series of linear equations derived from peak heights of unique fragments representative of each isomeric species. Several other approaches have also been proposed to deconvolute composite MS/MS spectra of peptide ions with different sequences^11^,^12^,^13^, however, their application is limited when analysing MS/MS spectra of co-fragmented isomeric ions that contain the same modification at different sites.

In this study, we present Iso-PeptidAce, a novel software tool that exploits high resolution LC–MS/MS data for deconvolution of composite spectra and quantification of site-specific acetylation. The resolution of composite MS/MS spectra derived from isomeric peptides is based on features associated with their characteristic fragment ion patterns obtained from the corresponding synthetic peptides and, importantly, does not rely on unique fragment ions to distinguish isomers. The application of Iso-PeptidAce is presented here for peptide isomers that contain multiple acetylated lysine residues but can also be applied to other types of modifications such as isomeric phosphopeptides^14^. The use of Iso-PeptidAce is demonstrated by monitoring temporal changes in acetylation patterns of histones H3 and H4 from human erythroleukemic (K562) cells treated with different classes of histone deacetylase inhibitors (HDACi), and by determining acetylation patterns in histones bound to *Saccharomyces cerevisiae* chromatin assembly factor-1 (CAF1).

## Results

### Deconvolution of mixed MS/MS spectra by Iso-PeptidAce

**Figure 1:**
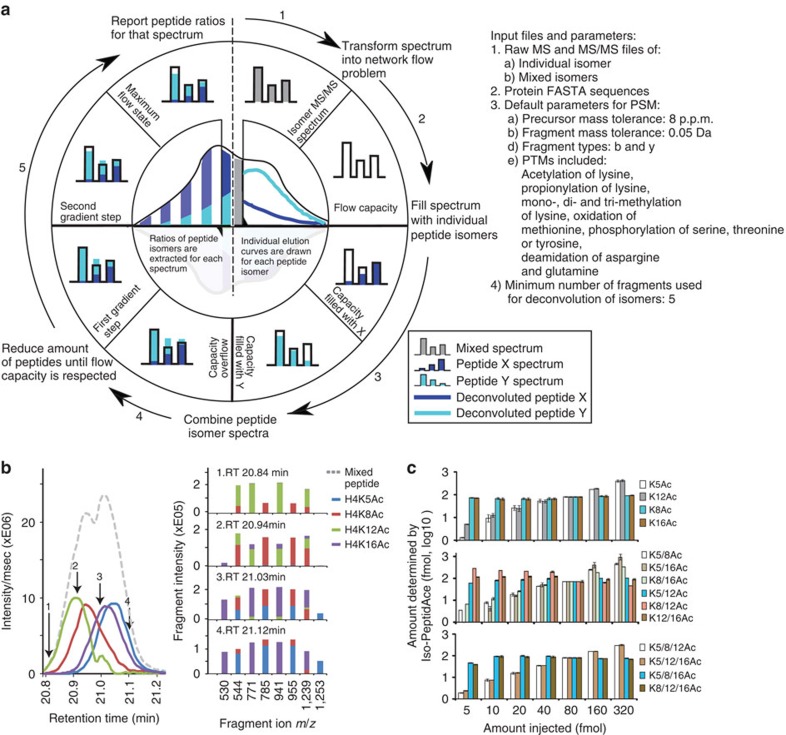
Deconvolution of co-eluting acetylated isomers by Iso-PeptidAce. (**a**) Schematic showing the process of spectral deconvolution by Iso-PeptidAce. The input files and parameters used for deconvolution of mixtures of isomers are listed on the right side of the panel. The process involves building an individual elution curve for X and for Y based on ratios computed for each MS/MS event. Isomeric peptide ratios are computed in five steps: (1) acquire and normalize fragment intensities of a composite spectrum; (2) model fragment intensities as a network flow with fixed capacities; (3) find the maximum flow for peptide X and for peptide Y; (4) combine both X and Y network flow capacities into the same network flow model; and (5) iteratively reduce the ratio of X and Y until the maximum flow is reached. (**b**) Left panel: elution profiles of a mixture of mono-acetylated isomers before (dashed line) or after (coloured lines) deconvolution. Right panel: the relative intensity of representative fragment ions extracted after isomer deconvolution at four different retention times, RT 20.84, 20.94, 21.03 and 21.12 min. (**c**) Bar graphs showing the amount of each peptide isomer determined by Iso-PeptidAce as a function of the amount of the same peptide injected as part of seven mixtures, each containing known amounts of the mono-, di- or tri-acetylated groups of isomers (from top to bottom). The mean value and the s.d. for each dilution point are shown in Supplementary Table 3A–C. The amount of peptide determined by Iso-PeptidAce in each mixture was normalized to the 80 fmol signal response. The amounts of peptides K5ac and K12ac (upper panel); K5_8ac, K5_16ac and K8_16ac (middle panel); K5_8_12ac and K5_12_16ac (lower panel) were varied from 5 to 320 fmol. Peptides K8ac and K16ac (upper panel); K5_12ac, K8_12ac and K12_16ac (middle panel); K5_8_16ac and K8_12_16ac (lower panel) were added to each of the seven mixtures at a fixed amount of 80 fmol. Each bar graph represents the mean of two technical replicates with error bars showing the relative distance of the maximum and the minimum values from the mean.

We designed a new software tool named Iso-PeptidAce that deconvolutes composite MS/MS spectra of known isomeric peptides. The software takes as input raw MS and MS/MS files of the individual and the mixed isomers, the FASTA sequence file of the protein of interest, and a set of parameters for peptide-spectrum matching (PSM) such as modifications, precursor and fragment ion tolerances (see Data analysis under the Methods section). The raw files are processed to extract precursor intensity, MS1 and MS2 injection times, and MS2 peak lists containing *m/z* and intensity values (see Supplementary Methods for a detailed section on spectral deconvolution and normalization of MS signal intensities). Iso-PeptidAce computes the proportion of individual isomers in composite MS/MS spectra based on fragment ion patterns that uniquely identify each isomer. In Iso-PeptidAce, fragment ion patterns are reduced to a set of maximum network flow problems, for which a number of efficient algorithms are known^15^. This approach has previously been used in a wide range of complex problems, such as predicting molecular pathways in complex diseases or selecting single nucleotide polymorphisms and their associated alleles in patient and control groups^16^,^17^,^18^. In our study, we implemented the network flow approach to deconvolute composite fragment spectra of acetylated isomers. Although these isomers share multiple fragment ions, they produce distinct fragment ion patterns that can be transformed into a set of network flow problems. Figure 1a shows a schematic overview of the deconvolution conducted by Iso-PeptidAce for a composite MS/MS spectrum generated from two hypothetical co-eluting isomeric peptides labelled X and Y. For every composite spectrum acquired across the elution curve, fragment ion intensities (Fig. 1a, step 1) are modelled into maximum network flow problems (flow capacity shown as empty bar charts, Fig. 1a, step 2). The networks are filled with fragment ion patterns for isomers X and Y (Fig. 1a, step 3) and merged into a single network with excess flow (represented as overrunning colour bars in Fig. 1a, step 4). The resulting network is processed iteratively by a multivariate optimization technique known as Gradient Descent^19^ (Fig. 1a, step 5), to remove the excess flow in the network. Each iteration step converges towards the maximum flow, which refers to the optimal ratio of X and Y compatible with the composite spectrum (Fig. 1a, left inner circle). Finally, individual elution curves for each isomer are generated based on the abundance ratios of MS/MS spectra (Fig. 1a, right inner circle) and the peak intensity or peak area of the precursor ions.

### Extraction of elution profiles and fragment ion patterns

We first used Iso-PeptidAce for MS/MS spectra of synthetic peptides from acetylated isomers of histones H3 and H4 (Supplementary Tables 1 and 2). All possible non-acetylated and acetylated forms of H4 peptide 1-SGRGKGGKGLGKGGAKRHR-19 were synthesized, and the purity of each peptide was confirmed by MS analyses. These peptides were propionylated, digested by trypsin and subjected to LC–MS/MS analysis on a Q-Exactive Plus instrument. We obtained three isomeric groups of H4 peptides acetylated at one, two or three lysine residues, and members of each group generally have very narrow retention time differences (Supplementary Fig. 1). To normalize precursor intensity, we determined the signal intensity response for each peptide as a function of the amount injected. We observed that mono- or multiply-acetylated isomers yielded a higher response than their propionylated counterparts (Supplementary Fig. 2A,B). We then prepared equimolar mixtures of peptides for each group of isomers and analysed them by LC–MS/MS. Although propionylated H4 peptides were readily separated into non-acetylated, mono-, di-, tri- and tetra-acetylated peaks (denoted as Ac0, 1, 2, 3 and 4 in Supplementary Fig. 3A, top panel), we observed that peptides within the same isomeric group co-eluted and produced mixed MS/MS spectra (Supplementary Fig. 3A,B). In addition, isomeric peptides shared multiple fragment ions (Supplementary Fig. 4A–E). Supplementary Fig. 4E shows a three-dimensional view of the abundance distribution of fragment ions derived from the four mono-acetylated isomers. H4K5ac and H4K16ac are the only mono-acetylated H4 peptide isomers that produced unique fragment ions at *m/z* 1253.7 and 530.3, respectively (Supplementary Fig. 4E, blue and purple bars). The peak intensities of these fragments can be used to infer the relative abundances of H4K5ac and H4K16ac in a given sample. This approach was previously implemented in existing software tools such as IsoScale^9^. Importantly, the two other mono-acetylated isomers, H4K8ac and H4K12ac did not produce any isomer-specific fragment ions (Supplementary Fig. 4E, red and green bars). These peptides share all of their fragments with either H4K5ac or H4K16ac (Supplementary Fig. 4E, blue and purple bars), and, as a result, fragment ion ratios cannot be used directly to measure the relative proportions of individual peptide isomers. In Iso-PeptidAce, deconvolution of composite MS/MS spectra containing fragments of the four mono-acetylated isomers is achieved by exploiting the distinguishing features associated with isomer-specific fragment ion patterns. Here the fragment ion pattern of a given isomer is defined by the retention time at which the peptide was eluted and selected for fragmentation, the peak height and *m/z* of each fragment ion, and the sites of modification. For example, the fragment ion pattern for H4K8ac is different from that of H4K12ac by the presence of additional fragmentions at *m/z* 785.5 and 955.6, and the absence of y-ion fragments at *m/z* 771.4 and 941.6 (Supplementary Fig. 4C,D) and b-ion at *m/z* 299.2. In addition, their shared fragments (for example, *m/z* 242.1, 544.3 and 1239.7) differ slightly in peak intensities (Supplementary Fig. 4C,D). Thus, by exploiting such isomer-specific differences, Iso-PeptidAce deconvoluted the co-eluting mono-acetylated peptides into four distinct elution profiles where the apex of individual isomer peaks were separated by a few seconds (Fig. 1b, left panel, coloured solid lines). The elution curve before the deconvolution is shown in Fig. 1b (grey dashed line). Using Iso-PeptidAce, we were able to determine the relative contribution of each isomer to the abundance of selected fragment ions (Fig. 1b, right panel). Most of these fragments are shared between the different isomers, but their distribution and proportion varies across peptides. Fragmentation patterns representative of H4K8ac and H4K12ac were more prominent at the beginning of elution profiles, whereas those of H4K5ac and H4K16ac were observed at later time points. Similarly, deconvolution of the di- and tri-acetylated isomers by Iso-PeptidAce enabled resolution of each isomeric peptide (Supplementary Fig. 5).

We then assessed the performance of Iso-PeptidAce using seven mixtures of peptides for each group of isomers (mono-, di-, tri-acetylated). Each peptide mixture contained known amounts of propionylated and acetylated isomeric H4 peptides each ranging from 5 to 320 fmols. Within each peptide mixture two to three peptides were selected and added to each mixture at a fixed amount of 80 fmols (Supplementary Table 2A–C). Raw MS and MS/MS files from these samples were submitted to Iso-PeptidAce for deconvolution. The amount of peptide determined by Iso-PeptideAce as a function of amount injected (in fmol) for the mono-, di- and tri-acetylated groups of isomers is shown in Fig. 1c (see Supplementary Table 3A–C for the mean values and error bars). We used the signal intensity observed at 80 fmols for normalization. In the seven mixtures of mono-acetylated peptides we observed an increase in the relative proportion of peptides H4K5ac and H4K12ac whereas peptides H4K8ac and H4K16ac (injected at a constant amount of 80 fmol in each mixture) remained unchanged in each mixture (Fig. 1c, upper panel and Supplementary Table 3A). Similarly, we observed increasing levels of H4K5ac/K8ac, H4K5ac/K16ac and H4K8ac/K16ac among the seven mixtures of di-acetylated peptides (Fig. 1c, middle panel and Supplementary Table 3C) and of H4K5ac/K8ac/K12ac and H4K5ac/K12ac/K16ac in the tri-acetylated mixtures (Fig. 1c, bottom panel and Supplementary Table 3B) in accordance to their expected abundance. Thus, Iso-PeptidAce successfully deconvoluted the composite spectra generated from different co-eluting acetylated isomers present at concentrations ranging over almost two orders of magnitude.

### Histone acetylation following HDAC inhibition

**Figure 2:**
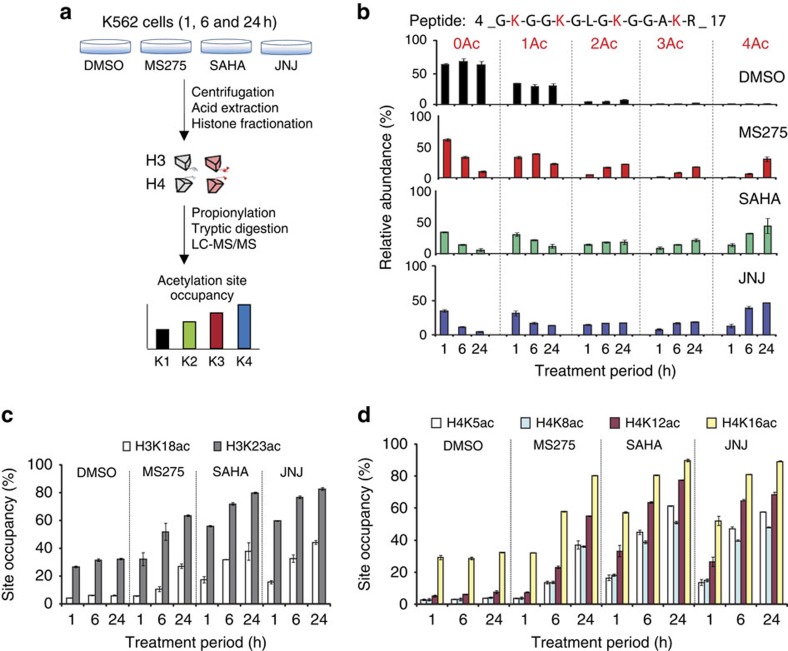
Analysis of acetylation site occupancies in histones H3 and H4 following HDACi treatment. (**a**) Histone isolation and MS analysis workflow. Total histones were isolated from human erythroleukemic (K562) cells treated for 1, 6 or 24 h with DMSO (control) or with the HDACi MS-275, SAHA and JNJ-26481585. Total histones were fractionated into individual core histones via offline RP-HPLC. Fractions containing histones H3 and H4 were subjected to propionylation, tryptic digestion and LC–MS/MS analysis. Acetylation site occupancy for each lysine residue was determined based on Iso-PeptidAce reported peptide intensities. (**b**) Relative abundance of unmodified (0Ac), mono- (1Ac), di- (2Ac), tri- (3Ac) and tetra-acetylated (Ac) peptide 4-GKGGKGLGKGGAKR-17 before (DMSO) or after treatment with MS-275, SAHA or JNJ-26481585. (**c**) Acetylation site occupancies of histone H3 at positions K18 and K23 and (**d**) histone H4 at positions K5, K8, K12 and K16. Each bar graph represents the mean of two technical replicates with error bars showing the relative distance from the mean of the maximum and minimum values.

We tested our software using tryptic digests of histones isolated from human K562 cells treated with HDAC inhibitors (Fig. 2a). Cells were treated in duplicate for 1, 6 or 24 h with dimethylsulphoxide (DMSO) as solvent control or with the HDAC inhibitors (HDACi) MS-275, SAHA and JNJ-26481585. Histones were isolated by acid extraction and fractionated by reverse phase liquid chromatography (Supplementary Fig. 6). Fractions containing histone H3 or H4 were subjected to propionylation, tryptic digestion and LC–MS/MS analysis before data deconvolution using Iso-PeptidAce (Fig. 2a). Our results show that, 24 h after treatment with SAHA or JNJ-26481585, ∼50% of H4 peptides 4-GKGGKGLGKGGAKR-17 were acetylated at the four available lysines, whereas only 20% of H4 molecules were tetra-acetylated in cells treated with MS-275 (Fig. 2b). Using Iso-PeptidAce we then determined acetylation site occupancies at H3K18, H3K23, H4K5, H4K8, H4K12 and H4K16 (Supplementary Table 4A,B). The abundance of H3K23ac is higher than that of H3K18ac in both untreated and HDACi-treated samples (Fig. 2c and Supplementary Table 4A). Although previous studies reported trace amounts of H3K18 methylation in mammalian cells^20^, we did not detect this modification in our samples. Cells treated with all three HDACi demonstrated similar increases in H4 acetylation as a function of time. We observed at least an eightfold increase in acetylation at H4K5, H4K8 or H4K12 after a 24 h treatment with MS-275, SAHA or JNJ (Fig. 2d and Supplementary Table 4B). Up to threefold increase in acetylation was observed at H4K16.

Although a number of studies have previously shown that the HDACi MS-275 and SAHA induce global increases in H3 and H4 acetylation, there was no efficient technique to measure site-specific acetylation stoichiometries of lysines in the N-terminal tail of H4 because some of the acetylated isomers cannot be resolved by nano-LC and do not generate isomer-specific fragments. In our study, we observed that both MS-275 and SAHA caused a major increase in acetylation of the four lysines in the N-terminal tail of H4. In addition, for the first time, we report site-specific changes in histone acetylation caused by the more recently characterized pan-HDACi JNJ-26481585 (ref. 21). This drug, also known as Quisinostat, is currently in phase II clinical trial and exhibits antitumour activity in human multiple myeloma and leukaemic cells^22^,^23^,^24^,^25^, and recent studies have shown that JNJ-26481585 elicits global increases in acetylation of histones H3 and H4 (refs 25, 26) However, owing to the inherent limitations of immunoassays, these studies did not report the specific lysine residues affected or the stoichiometries of acetylation. Our approach allowed identification of several H3 and H4 sites that increased in acetylation after JNJ-26481585 treatment. When compared with MS-275 and SAHA, JNJ-26481585 caused a more rapid and higher fold increase in acetylation at all the sites that we investigated (Fig. 2b–d). Consistent with these results, JNJ-26481585 has been reported to be 500-fold more potent than SAHA at inhibiting HDAC1 (ref. 21).

These examples illustrate how Iso-PeptidAce provides an automated and rapid approach to quantify global changes in acetylation site occupancies on histone lysine residues. Our method is implemented as a software tool that deconvolutes composite spectra derived from two or more co-eluting isomeric acetylated peptides. In turn, this enables an accurate assessment of acetylation site occupancies at each of the lysines within tryptic peptides.

### Acetylation patterns in CAF1-bound histones

**Figure 3:**
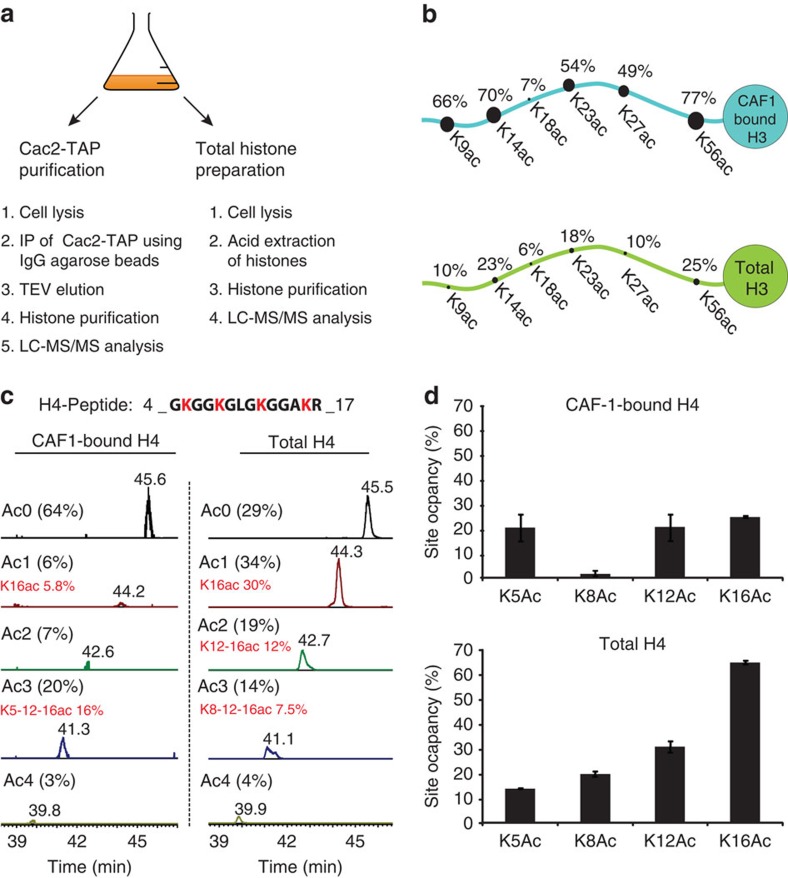
Analysis of acetylation site occupancies in CAF1 associated histones H3 and H4. (**a**) Schematic representation of the experimental workflow. Cac2-TAP was purified from 3.5 l of yeast cell culture and the remainder (0.5 l) was processed for total histone preparation. Histones from both samples were fractionated by offline RP-HPLC and analysed by LC–MS/MS. (**b**) Comparison of lysine acetylation site occupancies in total histone H3 (lower panel) and CAF1-bound H3 (upper panel). (**c**) Extracted ion chromatograms of histone H4 peptide 4-GKGGKGLGKGGAKR-17 acetylated at zero (Ac0), one (Ac1), two (Ac2), three (Ac3) or four (Ac4) lysine residues in CAF1-bound (left panel) or total histones (right panel). The predominant acetylation pattern in each group of isomers is highlighted in red. (**d**) Comparison of acetylation site occupancies at positions H4K5, H4K8, H4K12 and H4K16 in CAF1-bound H4 (upper panel) and total histone H4 (lower panel). Each bar graph represents the mean of two technical replicates with error bars showing the relative distance from the mean of the maximum and minimum values.

Acetylation of multiple residues in newly synthesized histones has been implicated in nucleosome assembly^27^. However, the molecular function and the biological implications of this acetylation have remained unclear because of redundancy among several acetylation sites in new H3 and H4 molecules. The capability of Iso-PeptidAce to determine acetylation patterns in situations where samples are limiting was evaluated for affinity-purified histones bound to CAF1, a protein complex that deposits new histone H3/H4 molecules onto nascent DNA during replication^28^. As previously described^28^, we purified the CAF1 complex from asynchronously growing yeast cells via a tandem affinity purification (TAP)-tagged Cac2 subunit. We also purified total histones from the same culture to compare their acetylation patterns with those of histones bound to CAF1 (Fig. 3a). The occupancies of H3/H4 acetylation sites were clearly different in CAF1-bound versus total histones (Fig. 3b–d and Supplementary Table 5). We also detected mono-, di- and tri-methylation of H3K36 in the total histones, but these PTMs were absent in CAF1-bound histones (Supplementary Table 5). These results argue that CAF1-bound histones did not dissociate and mix with other histones during our affinity purification procedure. Otherwise, the H3 and H4 acetylation patterns in CAF1-bound and total histones would be identical. In CAF1-bound H3 molecules, we found a high acetylation site occupancy (between 49 and 79%) at each of the five lysine residues previously known to be acetylated in new histones from *S. cerevisiae*: H3K56, K9, K14, K23 and K27, but not at K18 (Fig. 3b). This high degree of acetylation at multiple sites demonstrates that essentially all the H3 molecules bound to CAF1 are acetylated. In contrast, a large fraction (64%, Fig. 3c) of H4 molecules bound to CAF1 were not acetylated at any of the four lysine residues located in their N-terminal tail. Nonetheless, these results provide a potential explanation to a long-standing paradox. Hat1 is an enzyme that acetylates new H4 molecules at lysines 5 and 12, two sites of acetylation that are conserved from yeast to humans^27^. Given this, the paradox was that *S. cerevisiae* cells lacking Hat1 or cells where lysines 5 and 12 were mutated showed essentially no phenotype unless several lysines of H3 were also mutated to block their acetylation^29^,^30^. The fact that, in *S. cerevisiae*, the acetylation of new H3 molecules is far more abundant than that of new H4 molecules provides a molecular basis for these genetic results.

Our results also revealed an unexpected acetylation pattern in new H4 molecules where, in addition to lysines 5 and 12, lysine 16 was acetylated (Fig. 3c,d). Until now, the dogma was that new H4 molecules were predominantly acetylated at lysines 5 and 12, but not at lysine 16. This was based on pulse labelling of new histones with [^3^H]-lysine and Edman sequencing to quantify phenylthiohydantoin derivatives of acetyl-lysine and lysine^31^. The sensitivity of this technique rapidly decreases as a function of distance from the N-terminus and this may account for the fact that lysine 16 acetylation in new H4 molecules was not previously reported. Based on MS data, the presence of K16 acetylation in H4 molecules bound to *S. cerevisiae* CAF1 was previously reported^28^ but its prevalence was unknown. For the first time, our experiments demonstrate that the most abundant form of H4 bound to *S. cerevisiae* CAF1 consists of molecules that are tri-acetylated at K5, K12 and K16, with significantly lower amounts of mono- and di-acetylated forms that also contain K16 acetylation (Fig. 3c). Consistent with this, the acetylation site occupancies at K5, K12 and K16 were roughly 20% each in CAF1-bound H4, a pattern strikingly different from that observed in total histones (Fig. 3d). In *S. cerevisiae*, the integrity of heterochromatin depends on removal of H4K16 acetylation by the deacetylase Sir2 (ref. 32). Our results imply that Sir2-mediated deacetylation of H4K16 in new molecules deposited onto DNA during replication is likely important for propagation of heterochromatin structure in proliferating cells.

## Discussion

Iso-PeptidAce was initially created to solve a specific problem that had apparently been neglected, even though it was pointed out by Smith *et al.*^5^ more than 12 years ago. The problem of determining the relative abundance of multiple acetylated forms derived from the N-terminal tail of histone H4, which contains four acetylatable lysines, is compounded by the fact that several acetylated isomers co-elute during liquid chromatography and cannot be distinguished from each other based on the masses of MS^2^ fragments. The value of determining the relative abundance of acetylated isomers was illustrated by our studies of new histone molecules bound to CAF1. Unexpectedly, the predominant pattern of acetylation that we found consisted of H4 molecules tri-acetylated at K5, K12 and K16. Although further studies are necessary to assess its biological significance, the prevalence of this acetylation pattern has implications for both replication-coupled nucleosome assembly and the roles of histone deacetylases in the propagation of heterochromatin structures in proliferating cells.

It is known that in addition to lysine acetylation and methylation, histones are also modified by numerous other types of PTMs. Among many others, these include mono- or di-methylation of arginine, and several acylated forms of lysine residues that contain formyl, butyryl, crotonyl, malonyl or succinyl moieties. In the current study we did not determine the abundance of those types of PTMs because our method was developed with synthetic peptides carrying PTMs that were known to occur in yeast. Based on our data, we cannot rule out the possibility that other types of PTMs might also exist on the peptides that we studied. However, when coupled with recently developed multiplexed parallel reaction monitoring approaches^20^,^33^ we anticipate that our method will prove valuable for deconvolution of a large number of isomeric/isobaric peptides bearing numerous patterns of PTMs.

We also expect that our method will prove an asset to investigate a broad range of biological problems related to protein lysine acetylation. For instance, our approach will be valuable to determine the *in vivo* substrate specificity of the multitude of bromodomain (BRD)-containing proteins that exist in model organisms and human cells^3^. Previous *in vitro* studies have clearly shown that BRDs derived from chromatin-modifying enzymes bind with higher affinity to peptides that contain acetylated lysines located in close proximity^3^. Moreover, because large protein complexes contain multiple BRDs located in different subunits of a given complex, deciphering their physiologically relevant acetylated substrates remains a formidable task. Proteomics studies performed in several species have identified a myriad of acetylation sites in proteins with a wide range of biological functions^34^,^35^,^36^. Supplementary Table 6 shows seven examples of known acetylated peptides where isomers could now be distinguished based on their fragmentation patterns. We therefore anticipate that, when combined with techniques to co-purify protein complexes with their acetylated substrates, the method described here will help identify combinatorial patterns of lysine acetylation that are recognized by protein complexes involved in numerous biological processes.

## Methods

### Synthetic peptide preparations

Synthetic histone H3 and H4 peptides were purchased from GenScript and include four variants of the H3 peptide 17-RKQLATKAAR-26: one with unmodified lysines, two peptides with one acetylated lysine and one peptide with both lysines acetylated (Supplementary Table 1A). A total of 16 H4 peptides were purchased. These included one unmodified, four mono-acetylated, six di-acetylated, four tri-acetylated and one tetra-acetylated form of peptide 1-SGRGKGGKGLGKGGAKRHR-19 (Supplementary Table 1B). The concentration of each peptide was determined based on triplicate ultraviolet absorbance measurements at 205 nm using an Ultraspec 2100 Pro spectrophotometer (GE Healthcare). Each peptide solution was nominally in 1 μg μl^−1^ range. We verified the purity and identity of each peptide by injecting directly 1 pmol of each peptide on a Q-Exactive mass spectrometer (Thermo scientific). All MS files will be publicly available from PeptideAtlas database (data identifier: PASS00658, http://www.peptideatlas.org/PASS/PASS00658) For convenience MS/MS spectra of isomeric peptides are provided as Supplementary Fig. 7.

### Synthetic peptide mixtures

Standard curves were prepared for seven mixtures of H4 peptides with amounts of specific peptides ranging from 5 to 320 fmol (Supplementary Table 2A–C). Two or three peptides from each group of isomers were selected and added to the mixtures at a fixed amount of 80 fmols. The peptide mixtures were subjected to propionylation, tryptic digestion and LC–MS/MS analysis on a Q-Exactive mass spectrometer coupled to an EASY nLC II system (Thermo scientific).

### Detection efficiency of H3 and H4 acetylated peptides

We prepared five mixtures of H3 or H4 synthetic peptides. The amount of each peptide in mixtures 1, 2, 3, 4 and 5 are 25, 50, 100, 200 and 400 fmols, respectively. These samples were subjected to propionylation, tryptic digestion and LC–MS/MS analysis on a Q-Exactive Plus instrument. The signal intensity of each peptide was plotted against the amount of peptide injected as shown in Supplementary Fig. 2A,B.

### Cell treatment with HDAC inhibitors

Suspension cultures of K562 (human erythroleukemic) cells were grown in T-75 flasks at 37 °C and 5% CO_2_ in RPMI-1640 medium (Gibco) supplemented with 10% fetal bovine serum (Wisent) and 1% penicillin/streptomycin. Asynchronously growing cells were treated with 1 μM of SAHA, MS-275 or JNJ-26481585 (Selleck Chemicals) for 1, 6 and 24 h. Control cells were treated with DMSO. Two biological replicates were prepared for each condition. After treatment, the cells were collected, centrifuged at 1,000 r.p.m. at room temperature, washed with PBS, and flash frozen in liquid nitrogen. Histones were isolated using acid extraction as previously described^37^. Briefly, nuclei from 10^7^ cells were isolated with a hypotonic lysis buffer, followed by histone extraction using 0.2 M H_2_SO_4_ and TCA precipitation. The concentration of histone samples was determined by a Micro-BCA assay (Pierce). Further purification of core histones was performed by reverse-phase HPLC.

### Purification of yeast CAF1

The Cac2–TAP purification was adapted from Zhou *et. al*.^28^ Briefly, exponentially growing yeast cells (4 l at ∼1.8 × 10^7^ cells per ml in YPD medium) were collected by centrifugation and washed twice with ice-cold water. All further steps were carried out on ice. The pellet was resuspended in an equal volume of lysis buffer (25 mM Tris-HCl pH 7.5, 100 mM NaCl, 10 mM MgCl_2_, 0.1% NP-40, 1 mM EDTA, 10% glycerol, 1 mM DTT and 1 mM PMSF) containing a cocktails of protease inhibitors (5 μM Leupeptin, 25 μM Aprotinin and 5 μM Pepstatin A) and HDAC inhibitors (10 mM nicotinamide, 10 mM sodium butyrate, 10 μM trichostatin A and 100 μM SAHA). Cell suspensions were frozen in liquid nitrogen and lysed in a freezer mill (Spex Certiprep) using two repeats of four cycles with 2 min of grinding (15 impacts per second) and 2 min of cooling per cycle. The resulting cell lysate powder was thawed out on ice. Ethidium bromide (75 μg ml^−1^) was added and the lysate was incubated on ice for 1 h with benzonase (25 U ml^−1^). The extract was clarified by centrifugation at 25,000 r.p.m. for 30 min. The supernatant was then incubated with 200 μl of IgG-Agarose beads (SIGMA) for 2 h at 4 °C. The beads were recovered by centrifugation, extensively washed with lysis buffer followed by several washes with TEV cleavage buffer (10 mM Tris-HCl pH 8, 100 mM NaCl, 1 mM DTT and 0.5 mM EDTA) containing the protease inhibitor and HDAC inhibitor cocktails. To elute Cac2–TAP, the beads were incubated with TEV protease at 4 °C overnight in TEV cleavage buffer. A portion of the TEV eluate (30 μl) was resolved by SDS–PAGE and the presence of Cac2-CBP and histones H3 and H4 was confirmed by immunoblotting. The remainder of the TEV eluate (170 μl) was dried in a Speed-Vac, resuspended in 1 ml H_2_O and applied to a C18 column equilibrated with 0.1% trifluoroacetic acid (TFA) in water. The C18 column was washed twice with 1 ml of 0.1% TFA in 5% methanol and eluted with 1 ml of 70% acetonitrile (ACN). The eluate was dried in a Speed-Vac and resuspended in 0.1% TFA for purification of intact histones by offline HPLC.

### Fractionation of core histones by RP-HPLC

Separation of intact core histones was achieved using a narrow-bore Zorbax C8 reverse-phase column (2.1 × 150 mm, 5 μm, 300 Å) on an Agilent 1200 HPLC system. Solvent A was aqueous 0.1% TFA (Sigma) and solvent B was 0.1% TFA in 100% acetonitrile (ACN). Approximately 2 μg of acid extracted histones were loaded onto the C8 column at a flow rate of 150 μl min^−1^. Histones were eluted from the column using a gradient of 5–80% solvent B in 60 min. Fractions were collected in a 96-well plate at a rate of one fraction per minute. Fractions containing histones H3 or H4 were pooled and dried completely in a Speed-vac concentrator.

### Propionylation and trypsin digestion of histones

In-solution tryptic digestion of synthetic peptides or core histones was performed as previously described^38^. Briefly, a total of 2 μg of HPLC purified histones H3 and H4 were subjected to propionylation by adding 200 μl of freshly prepared 2:1 (v/v) water: propionic anhydride (Sigma) mixture and vortexing the mixture for 1 h at room temperature. The samples were then dried in a Speed-vac at 4 °C. The dried samples were resuspended in 50 mM ammonium bicarbonate, vortexed for 2 min and subjected to a second round of evaporation at 4 °C. The samples were collected and resuspended in 100 μl of 50 mM ammonium bicarbonate and vortexed for 5 min to re-dissolve the proteins. Our digestion solution was prepared by adding 200 μl of 50 mM ammonium bicarbonate in a vial containing 20 μg of lyophilized trypsin (Promega). Roughly 0.5 μl of this solution was added to each histone sample and digested overnight at 37 °C. After digestion, samples were dried completely in a Speed-vac and then resuspended in 0.2% formic acid before LC–MS/MS analyses.

### LC–MS/MS analyses of histone digests

MS data were acquired in duplicate on a Q-Exactive Plus mass spectrometer (Thermo scientific) coupled to an EASY nLC II system (Thermo scientific). A total of 1 μg of histone H3 and H4 digests generated from the control or HDACi-treated cells were first desalted on a Jupiter C18 (3 μm particles, Phenomenex) trap column (4 mm length, 360 μm i.d.) for 5 min at 10 μl min^−1^, before their elution onto a C18 analytical column (18 cm length, 150 μm i.d.). A linear gradient from 5 to 60% ACN (containing 0.2% formic acid) at 600 nl min^−1^ over 60 min was used for peptide elution. The MS instrument was operated in positive ion mode and capillary voltage of 1.6 kV. MS scans were acquired in the Orbitrap analyser over the range of 300–1,500 *m/z* at a resolution of 70,000 and automatic gain control target value of 1.0 × 10^6^. An inclusion list containing *m/z*, charge state and collision energy values of H3 and H4 peptides was used to trigger MS/MS acquisition. Every precursor ion found in the inclusion list was automatically selected for fragmentation in the HCD cell at a normalized collision energy setting of 27. The fragments were analysed in the Orbitrap at a resolution of 35,000 and a target value of 5.5 × 10^5^. The dynamic exclusion setting was disabled to acquire multiple MS/MS spectra per peptide.

### Data analysis

Data analysis was performed using the Iso-PeptidAce software. Raw MS and MS/MS files of individual synthetic peptides, mixtures of isomeric peptides or histone digests and Fasta files with H3 and H4 protein sequences were submitted to Iso-PeptidAce. The default settings for deconvolution of composite MS/MS spectra are: precursor mass tolerance: 8 p.p.m., fragment mass tolerance: 0.05 Da, the minimum number of fragment ions (per isomer) considered for deconvolution: 5, types of fragment ions considered: b and y, digestion enzyme: trypsin, missed cleavages: Iso-PeptidAce uses a no-enzyme *in silico* protein digestion routine to parse the provided FASTA files for potential matches. PTMs included in peptide-spectrum matching: carbamidomethylation of cysteine (C, 57.0215 Da), oxidation of methionine (M, 15.9949), phosphorylation of serine, threonine or tyrosine (S/T/Y, 79.9663), deamidation of asparagine and glutamine (N/Q 0.9840/0.9847), acetylation of lysine (K, 42.0106), propionylation of lysine (K, 56.0262), mono-methylation of lysine (K, 14.0157), di-methylation of lysine (K, 28.0313), tri-methylation of lysine (K, 42.0470), acetylation of protein N-terminus (42.0106). All the PTMs were considered as variable modifications. The output from Iso-PeptidAce is a combined result file (spreadsheet) containing intensity values for all the acetylated and non-acetylated forms of the H3 and H4 peptides obtained after deconvolution. A representative result file for the H4 peptide GKGGKGLGKGGAKR is shown in Supplementary Table 7. A total of 16 different isoforms of this peptide were detected (shown in each column in Supplementary Table 7). These peptides are divided into five groups (per sample) based on their *m/z* values (shown in each row in Supplementary Table 7): one un-acetylated (0Ac, *m/z* 747.94), four mono-acetylated (1Ac, *m/z* 740.93), six di-acetylated (2Ac, 733.93), four tri-acetylated (3Ac, *m/z* 726.92) and one tetra-acetylated (4Ac, *m/z* 719.91). Each intensity value in Supplementary Table 7 is normalized based on the peptide's response factor, which is determined from the slope of the lines shown in Supplementary Fig. 2A,B. The slope of each line was determined from the linear equations that best fit the MS signal responses. Acetylation site occupancy at a specific Lys residue was determined from the ratio of the sum of intensities of peptides bearing the ac-Lys to the sum of intensities of all the 16 peptide isoforms. Iso-PeptidAce can be downloaded from the website: http://proteomics.iric.ca/tools/Iso-PeptidAce. Detailed information on Iso-PeptidAce is also provided as Supplementary Methods.

## Additional information

**Accession codes.** MS files are available from PeptideAtlas database (data identifier: PASS00658).

**How to cite this article:** Abshiru, N. *et al.* Discovery of protein acetylation patterns by deconvolution of peptide isomer mass spectra. *Nat. Commun.* 6:8648 doi: 10.1038/ncomms9648 (2015).

## Supplementary Material

Supplementary Information Supplementary Figures 1-7, Supplementary Tables 1-7, Supplementary Methods and Supplementary Reference
