# Supplementary material for: Discovery of protein acetylation patterns by deconvolution of peptide isomer mass spectra
Source: Nat Commun. 2015 Oct 15;6:8648. doi: 10.1038/ncomms9648 (PMC4667697; doi:10.1038/ncomms9648)
Supplement: Supplementary Information — Supplementary Figures 1-7, Supplementary Tables 1-7, Supplementary Methods and Supplementary Reference [file ncomms9648-s1.pdf]

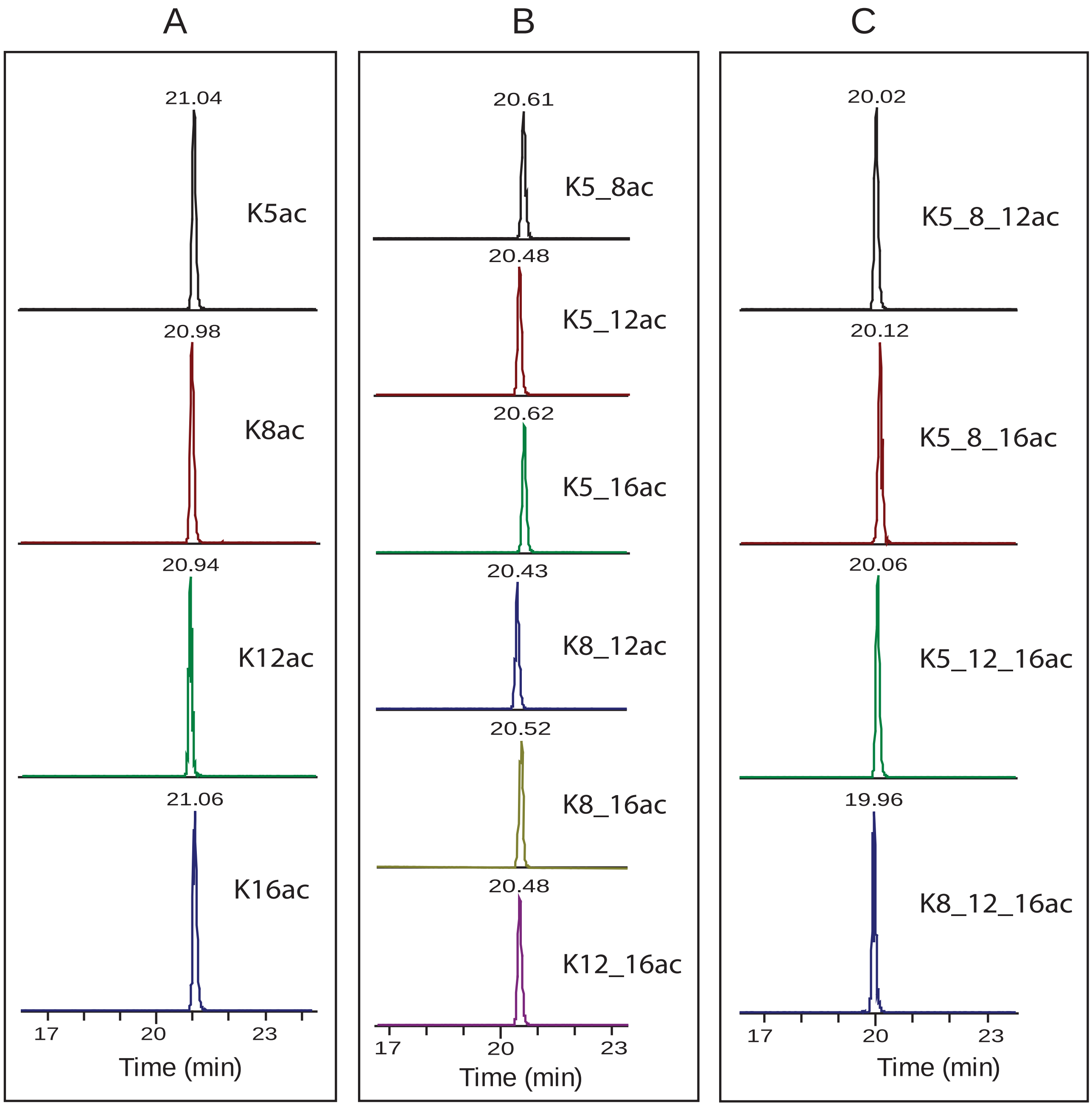

Supplementary Figure 1: Peptides within the same isomeric groups exhibit very narrow retention time differences. After in vitro propionylation and trypsin digestion of our synthetic peptides, we assessed their m/z ratio, charge state and retention time by LC-MS analysis. Shown in this figure are the extracted ion chromatograms of doubly charged isomers of (A) mono-acetylated and triply propionylated (m/z 740.93), (B) di-acetylated and di-propionylated (m/z 733.93), and (C) tri-acetylated and mono-propionylated (m/z 726.92) H4 peptide 4-GKGGKGLGKGGAKR-17

A) H3 peptide: 18-**K**QLAT**K**AAR-26

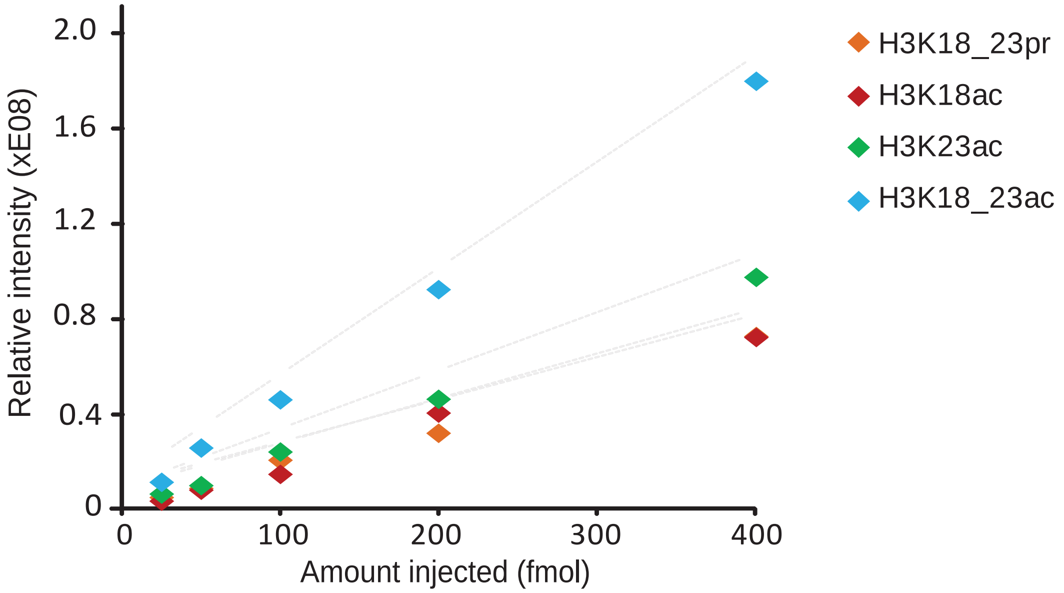

Signal response	Slope
K18_23pr	183967
K18ac	190984
K23ac	251676
K18_23ac	460783

B) H4 peptide : 4-G**K**GG**K**GLG**K**GGA**K**R-17

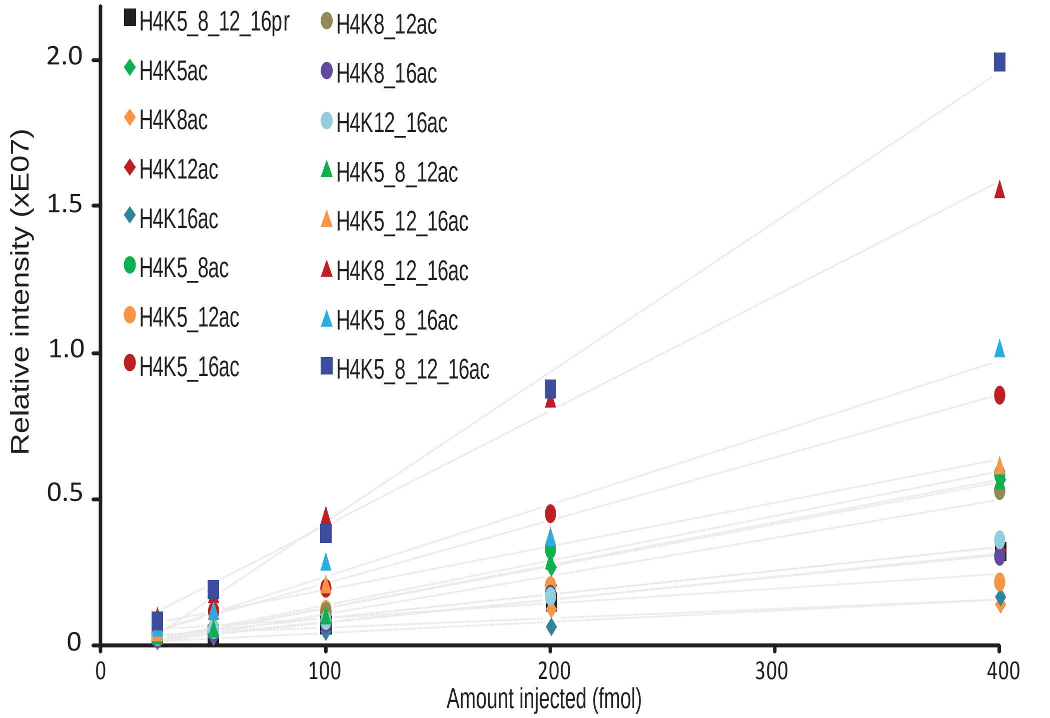

Signal response	Slope
K5/8/12/16pr	7898.8
K5Ac	14331
K8Ac	3181.5
K12Ac	8033.1
K16Ac	3818.7
K5/8Ac	15163
K5/12Ac	5051.5
K5/16Ac	21465
K8/12Ac	13017
K8/16Ac	7617.1
K12//16Ac	8762.5
K5/8/12Ac	14529
K5/8/16Ac	14799
K5/12/16Ac	39046
K8/12/16Ac	24572
K5/8/12/16Ac	51021

Supplementary Figure 2: MS signal responses of unmodified and acetylatied H3 and H4 peptides. After in vitro propionylation and trypsin digestion of synthetic peptides, the relative MS response of each peptide was determined based on the curve of ion intensity versus amount injected for the non-acetylated and acetylated forms of (A) H3 peptide 18-KQLATKAAR-26 and (B) H4 peptide 4-GKGGKGLGKGGAKR-17. The tables on the right show the slopes of the lines determined from the linear equation that best-fit (as measure by R2 values) the signal responses.

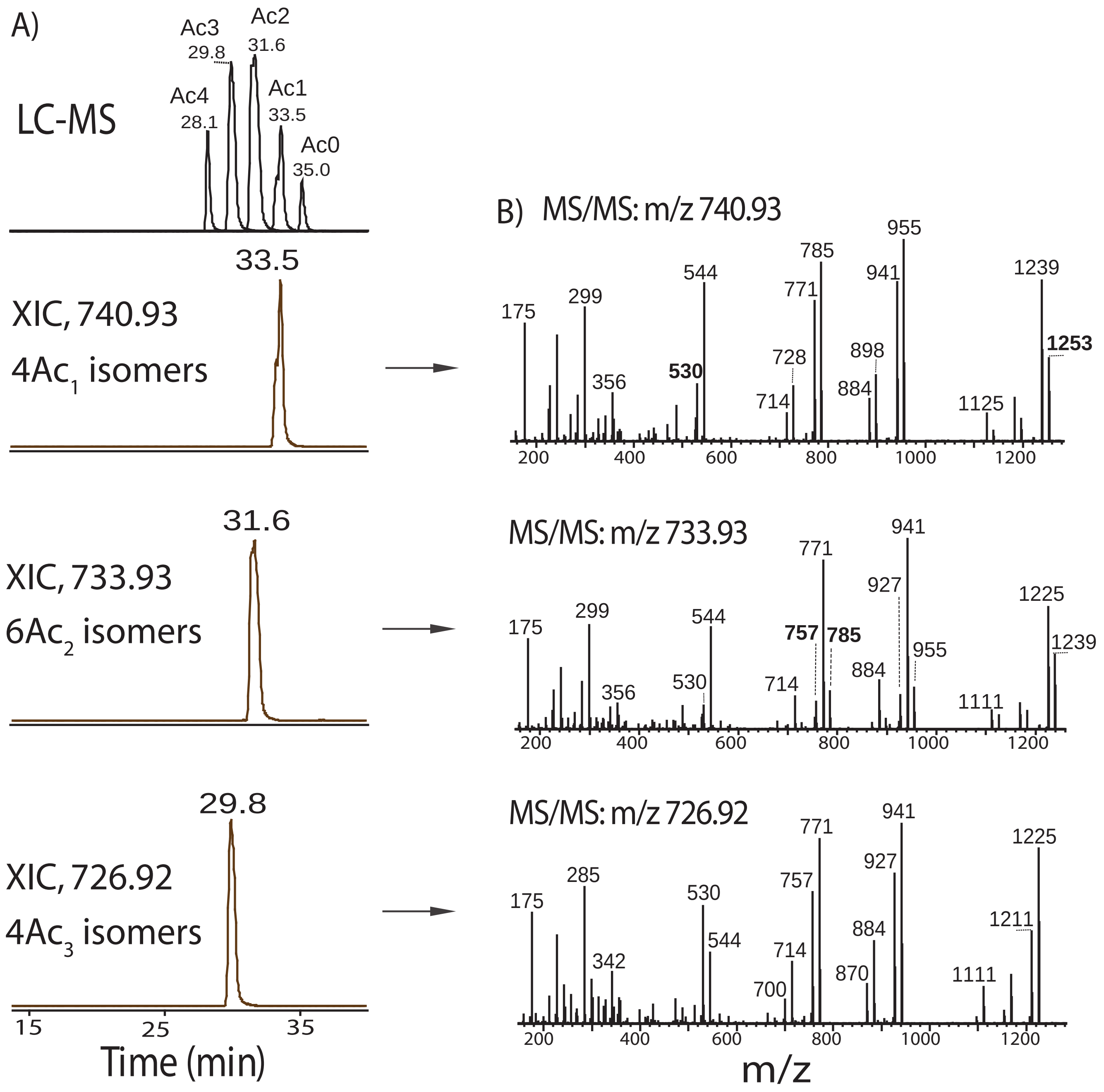

Supplementary Figure 3: Acetylated isomers co-elute from reverse-phase column and produce composite MS/MS spectra upon fragmentation. A) LC-MS trace of a mixture of sixteen H4 peptides, and extracted ion chromatograms of co-eluting four mono-, six di- and four tri-acetylated isomers of peptide 4-GKGGKGLGKGGAKR-17 (top to bottom panels). B) Mixed MS/MS spectra of four mono-, six di- and four tri-acetylated isomers (from top to bottom panel).

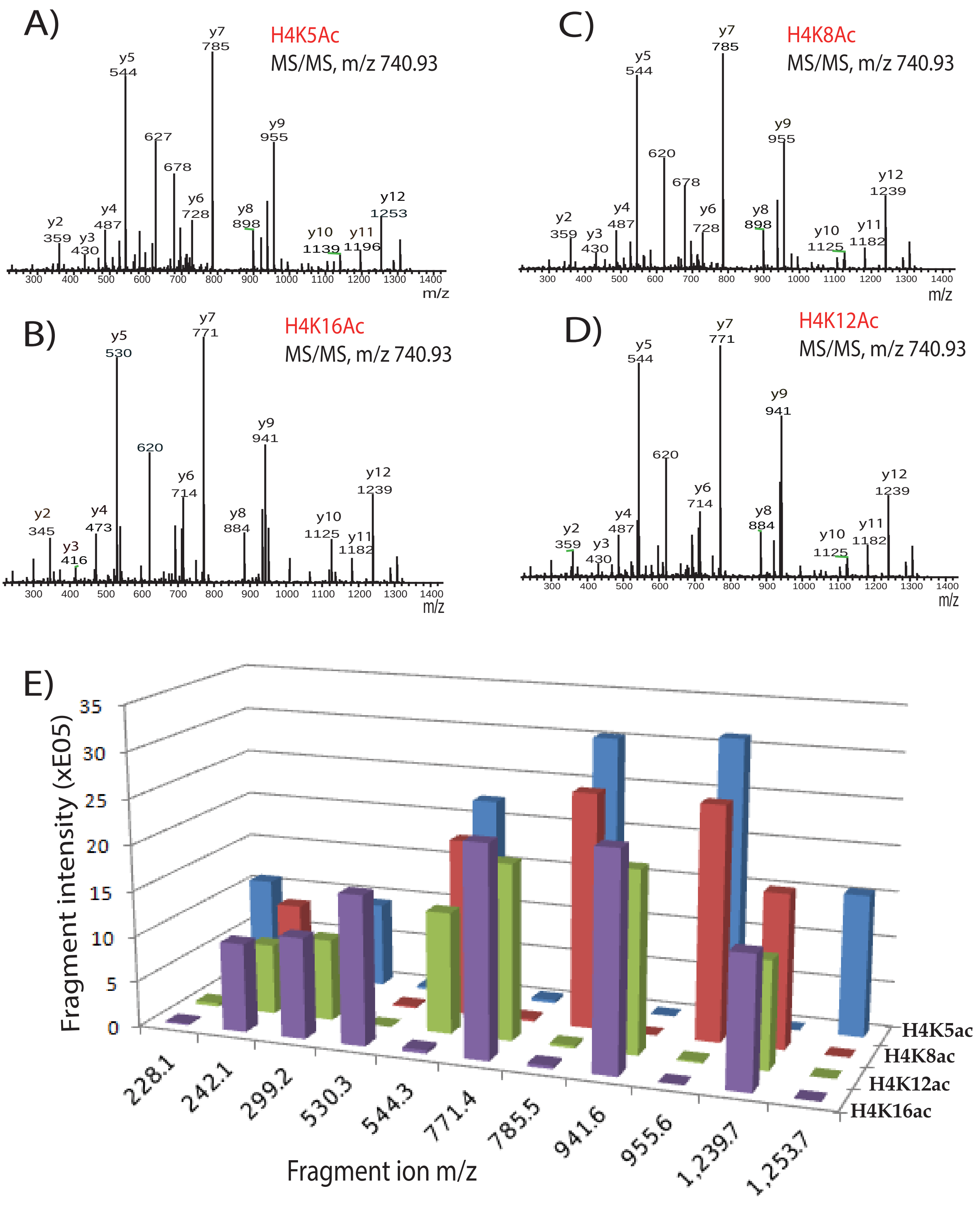

Supplementary Figure 4: Isomeric peptides produce distinct fragment ion patterns upon fragmentation in the MS. Representative fragment ion spectra of four mono-acetylated isomers ofthe peptide 4-GKGGKGLGKGGAKR-17: (A) H4K5ac, (B) H4K16ac, (C) H4K8ac, and (D) H4K12ac. The ‘y’ type fragment ions are indicated in each spectrum. E) A three dimensional view of the distribution of selected ‘b’ and ‘y’ type fragment ions derived from the individual mono-acetylated isomers. The ‘b’ type fragment ions are shown at m/z 228.1 (b2, K5ac), 242.1 (b2, K5pr) and 299.2 (b3, K5pr). The ‘y’ type fragment ions are m/z 530.3, 544.30, 771.4, 785.5, 941.6, 955.6, 1239.7 and 1253.7.

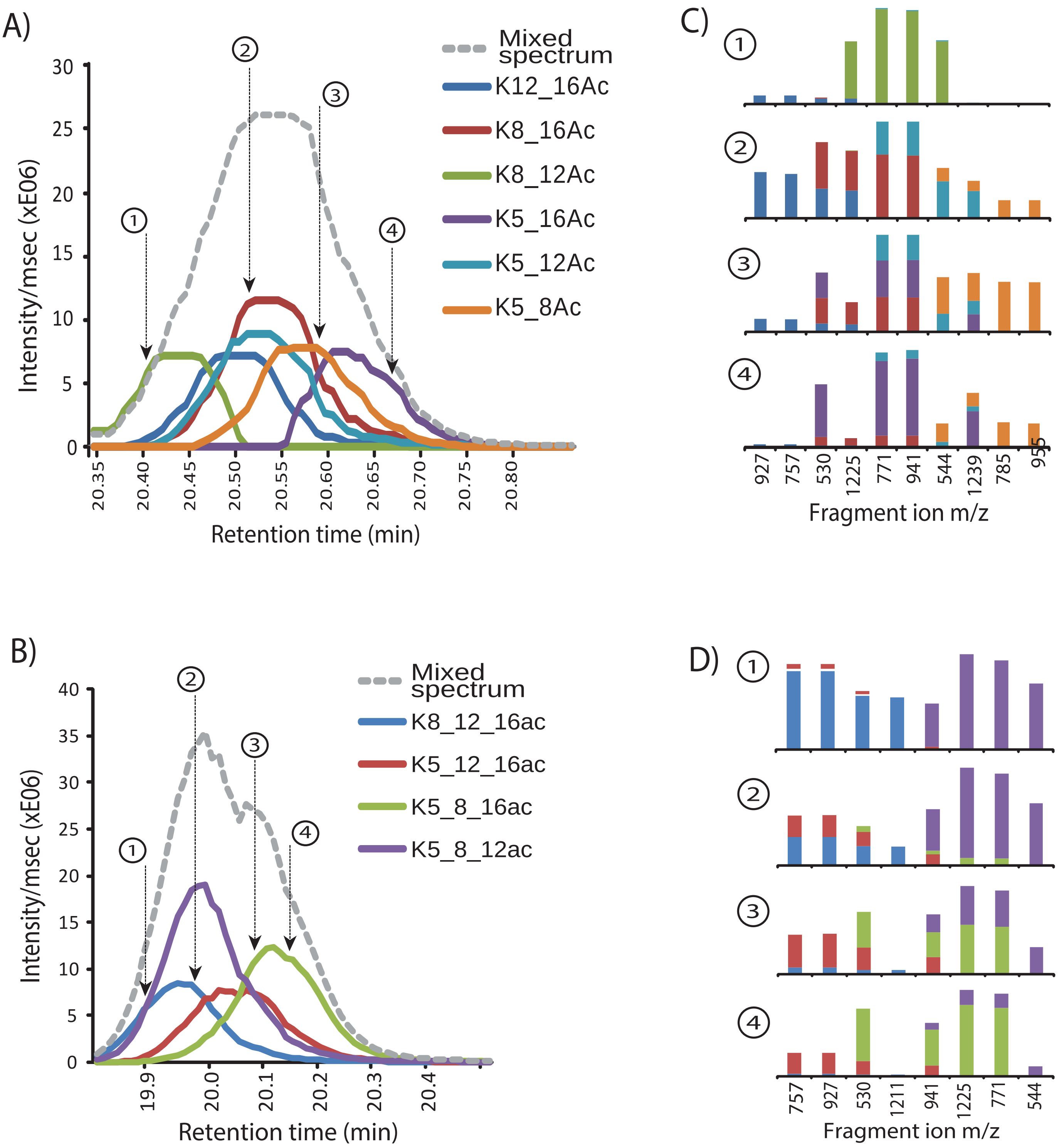

Supplementary Figure 5: Deconvolution of co-eluting di- and tri-acetylated isomers of histone H4 peptides by Iso-PeptidAce. Elution profiles before (dashed line) or after (colored lines) deconvolution of a mixture of (A) six di-acetylated, and (B) four tri-acetylated isomers of the peptide 4-GKGGKGLGKGGAKR-17. Distributions of fragment peaks of the deconvoluted di- (C) and tri-acetylated (D) isomers.

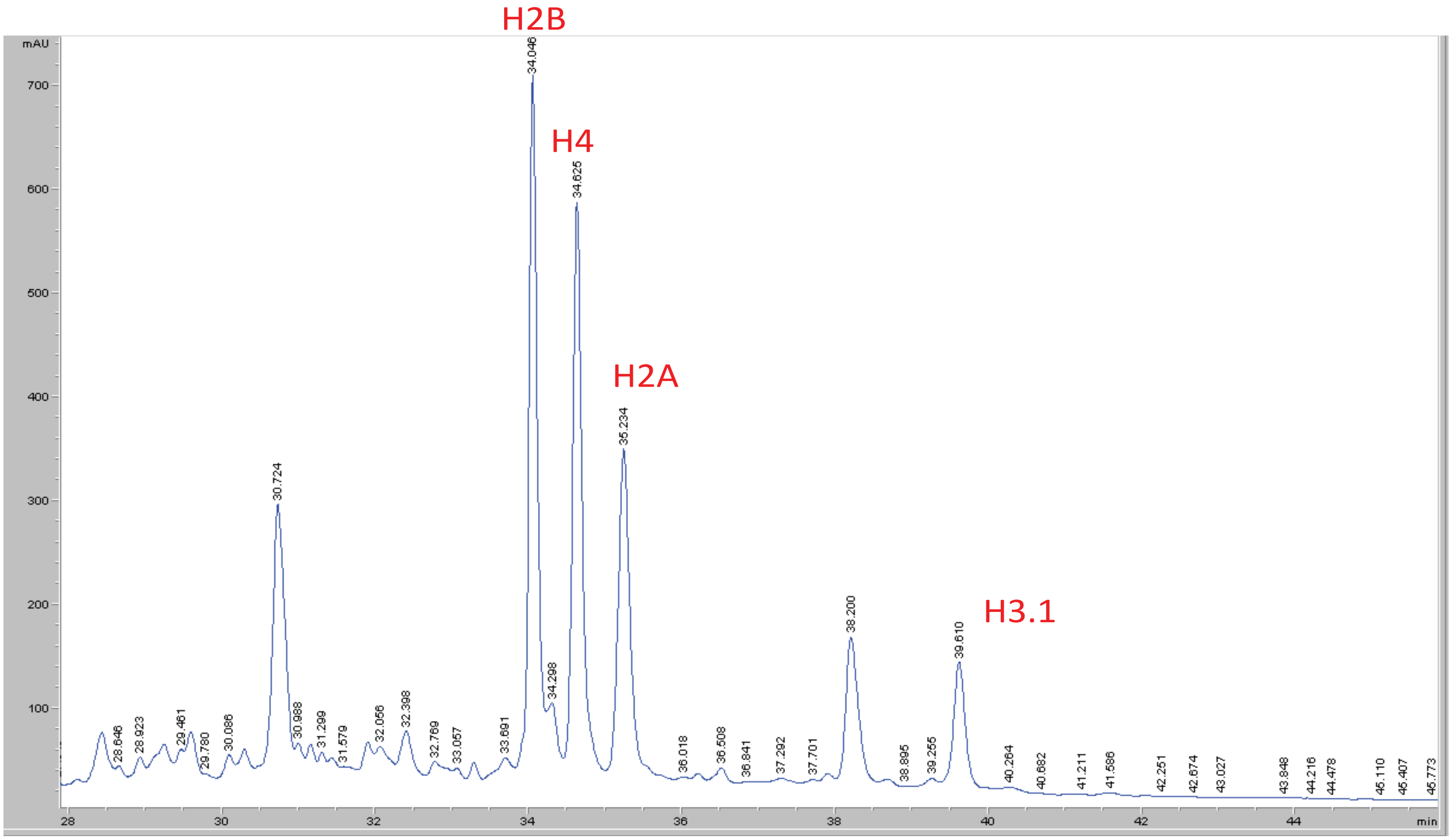

Supplementary Figure 6: Fractionation of total histones by RP-HPLC. Total histones were baseline separated as H2B, H4, H2A and H3.1 in increasing order of retention time. Histone H3.1 and H4 fractions were pooled and dried completely in a speed-vac concentrator. LC conditions: Pump A: 0.1% trifluoroacetic acid in 100% filtered water (v/v). Pump B: 0.1% trifluoroacetic acid in 100% acetonitrile (v/v)., flow rate:150µl/min, Absorbance wavelength: 214 nm, Column: Zorbax C8, 5µm, 2.1x150 mm

Supplementary Figure 7 - MS/MS spectra of peptide isomers

Peptide-1: H3K18pr_K23pr
K(pr)QLATK(pr)AAR

MS/MS 549.84^{2+}

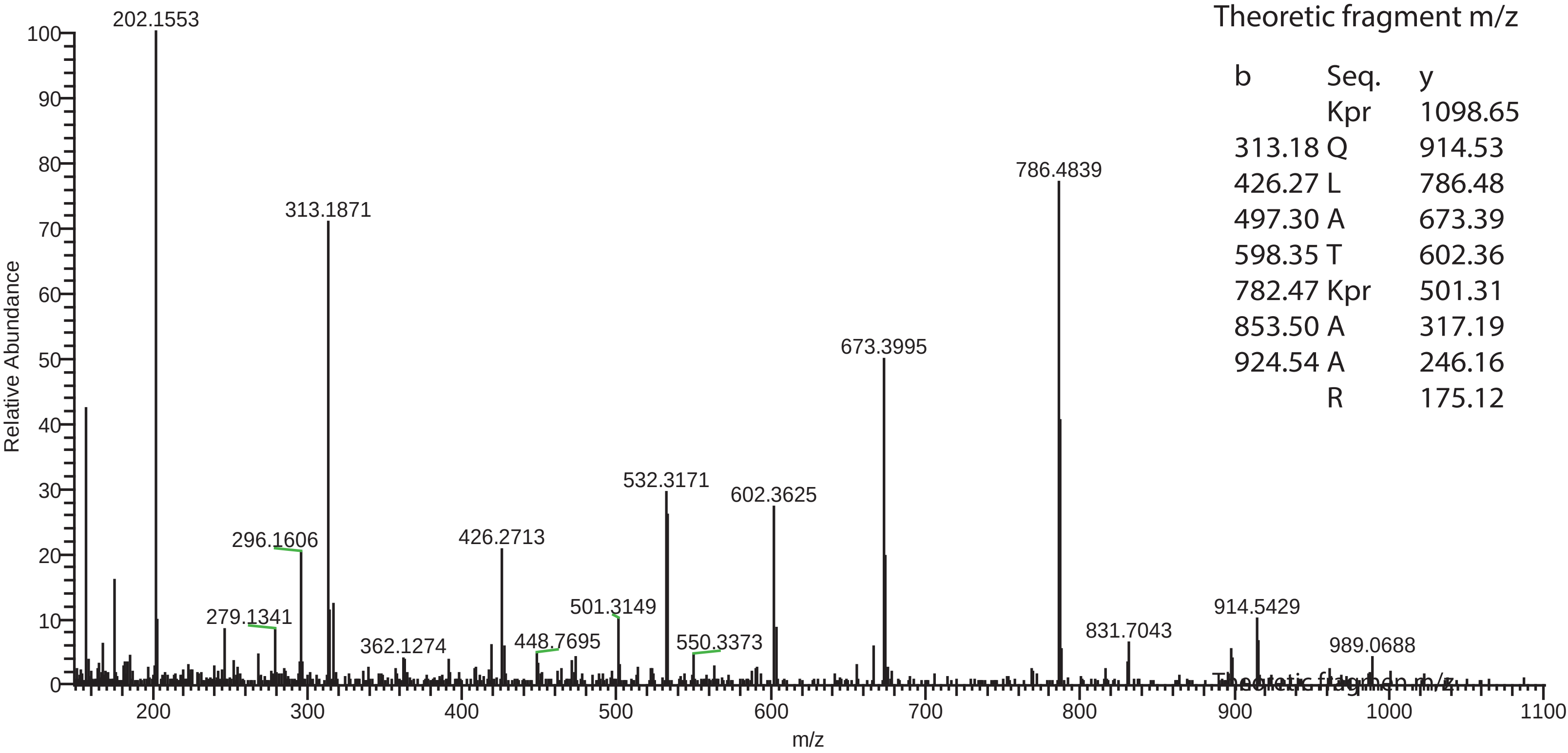

Theoretic fragment m/z

b	Seq.	y
	Kpr	1098.65
313.18	Q	914.53
426.27	L	786.48
497.30	A	673.39
598.35	T	602.36
782.47	Kpr	501.31
853.50	A	317.19
924.54	A	246.16
	R	175.12

Peptide-2: H3K18ac_K23pr
K(ac)QLATK(pr)AAR

MS/MS 542.83^{2+}

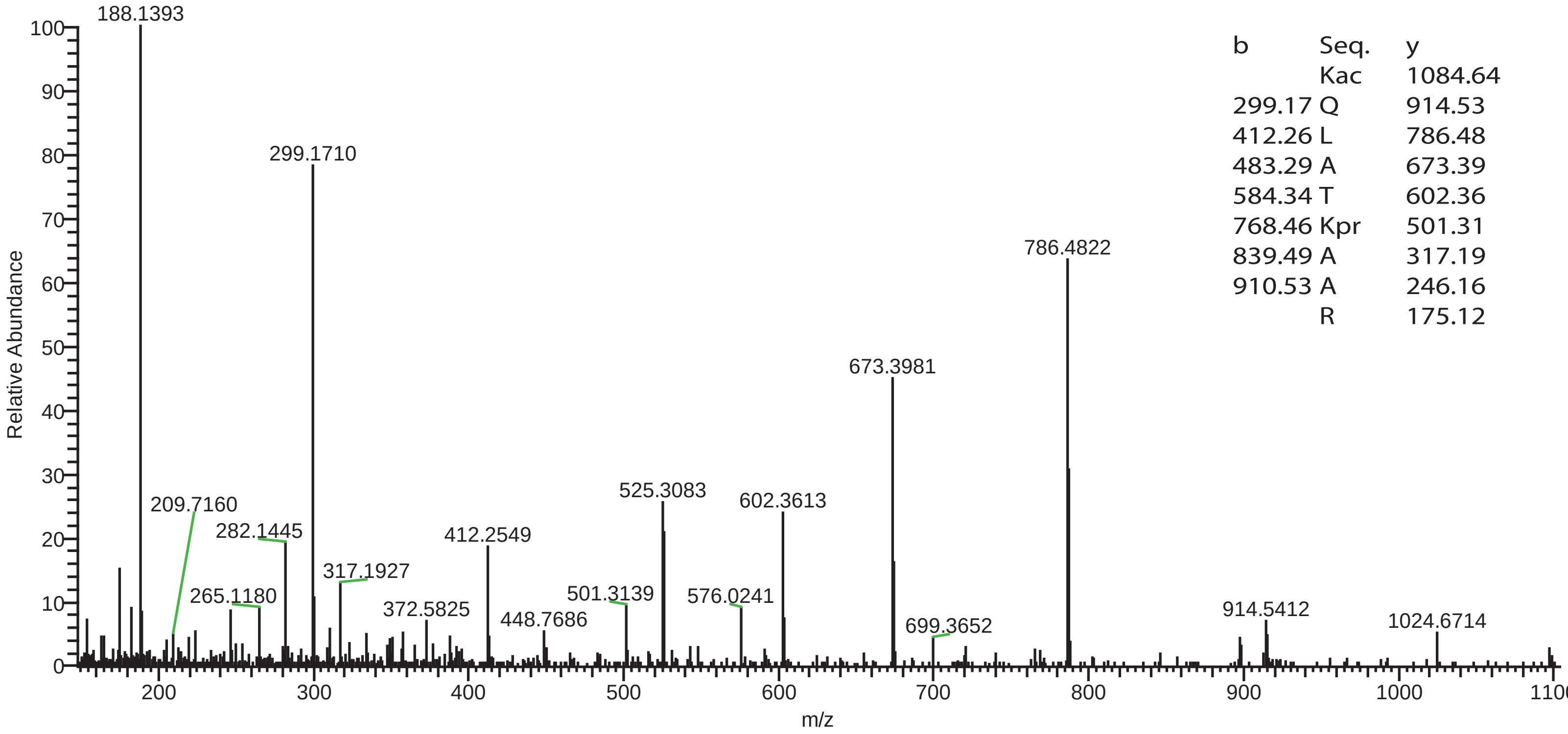

b	Seq.	y
	Kac	1084.64
299.17	Q	914.53
412.26	L	786.48
483.29	A	673.39
584.34	T	602.36
768.46	Kpr	501.31
839.49	A	317.19
910.53	A	246.16
	R	175.12

Peptide-3: H3K18pr_K23ac
K(pr)QLATK(ac)AAR

MS/MS 542.83^{2+}

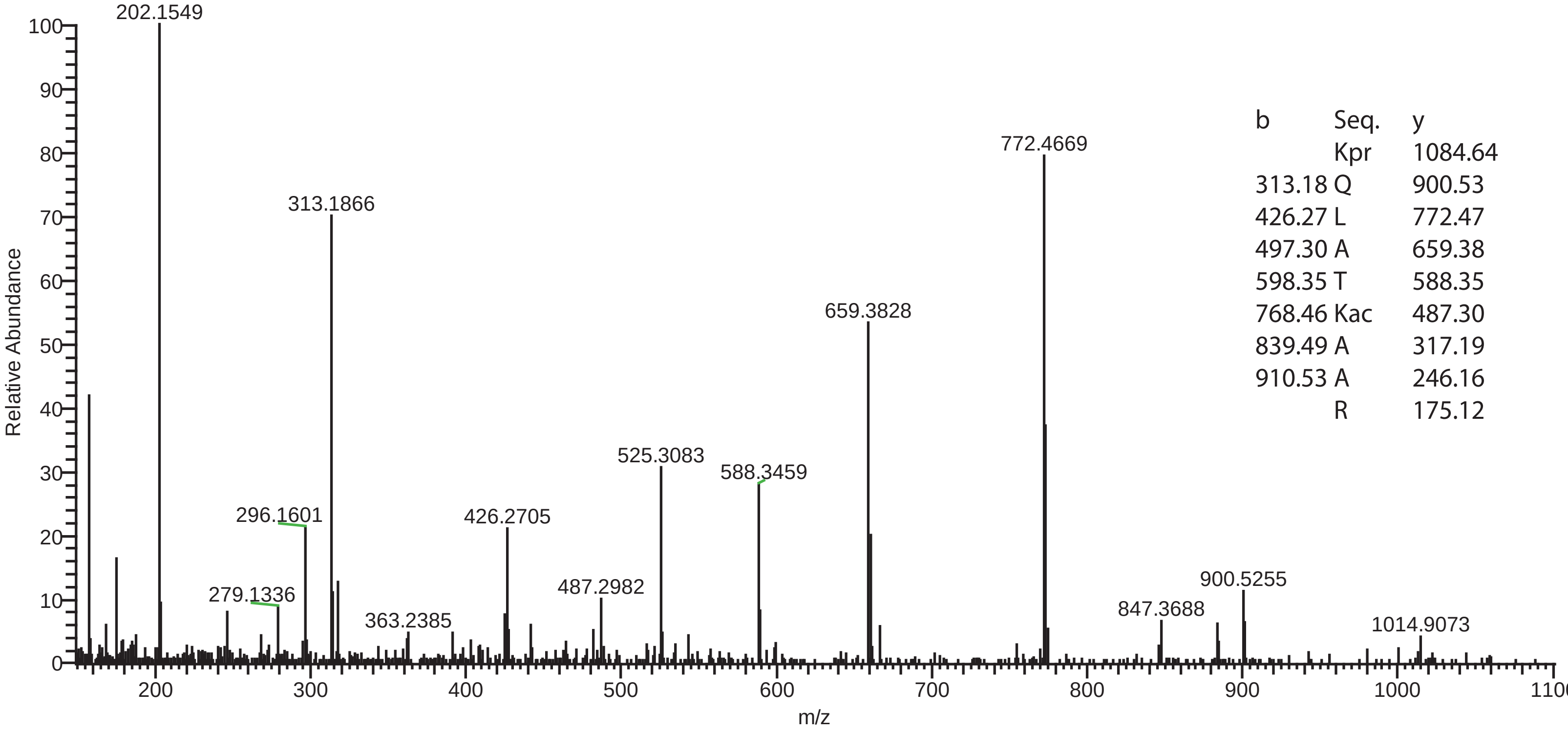

b	Seq.	y
	Kpr	1084.64
313.18	Q	900.53
426.27	L	772.47
497.30	A	659.38
598.35	T	588.35
768.46	Kac	487.30
839.49	A	317.19
910.53	A	246.16
	R	175.12

Peptide-4: H3K18ac_K23ac
K(ac)QLATK(ac)AAR

MS/MS 535.82^{2+}

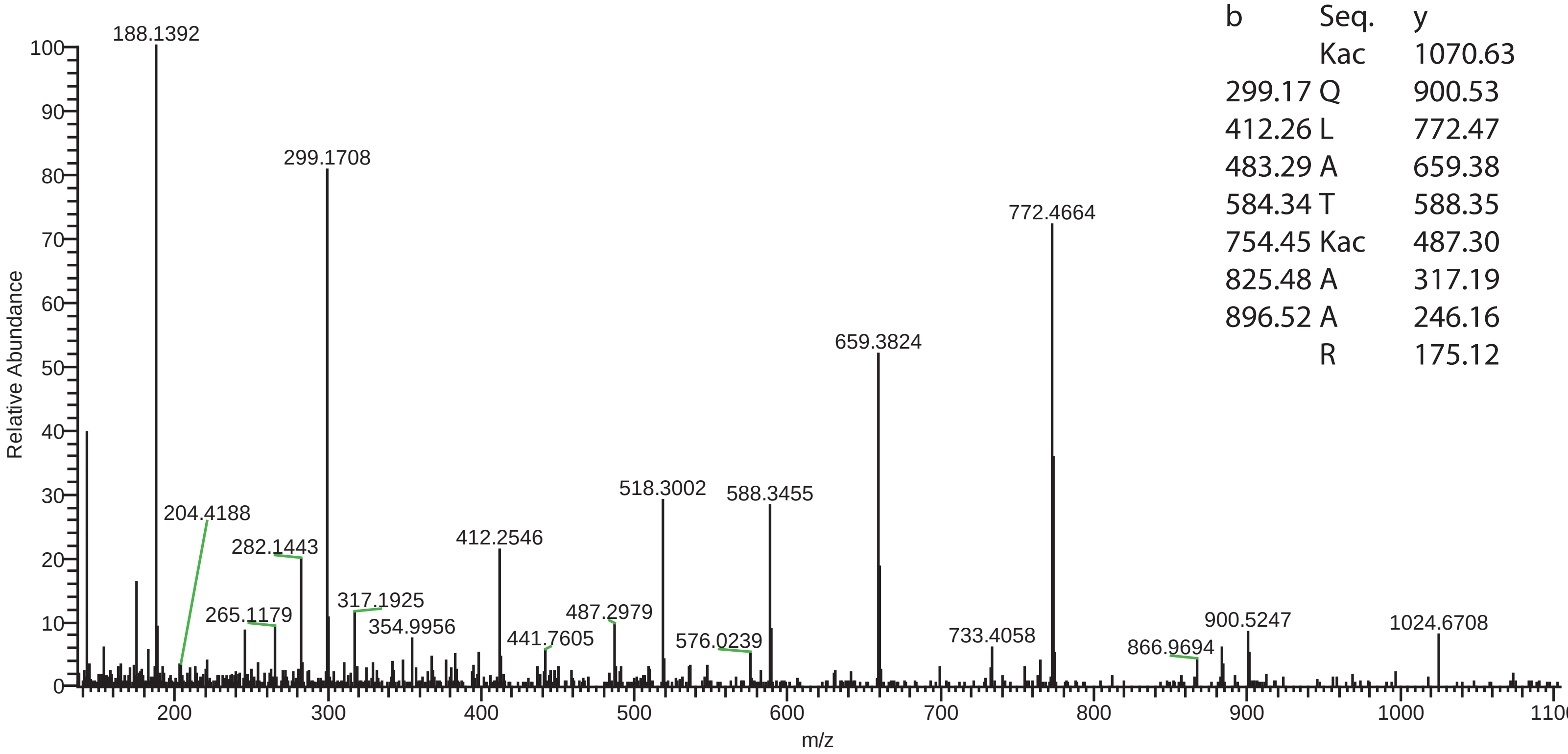

b	Seq.	y
	Kac	1070.63
299.17	Q	900.53
412.26	L	772.47
483.29	A	659.38
584.34	T	588.35
754.45	Kac	487.30
825.48	A	317.19
896.52	A	246.16
	R	175.12

Peptide-5: H4K5_8_12_16pr
GK(pr)GGK(pr)GLGK(pr)GGAK(pr)R

MS/MS 747.94^{2+}

b	Seq.	y
	G	1494.85
242.14	Kpr	1437.83
299.17	G	1253.71
356.19	G	1196.69
540.30	Kpr	1139.67
597.32	G	955.56
710.41	L	898.53
767.43	G	785.45
951.54	Kpr	728.43
1008.5	G	544.31
1065.59	G	487.29
1136.62	A	430.27
1320.74	Kpr	359.23
	R	175.12

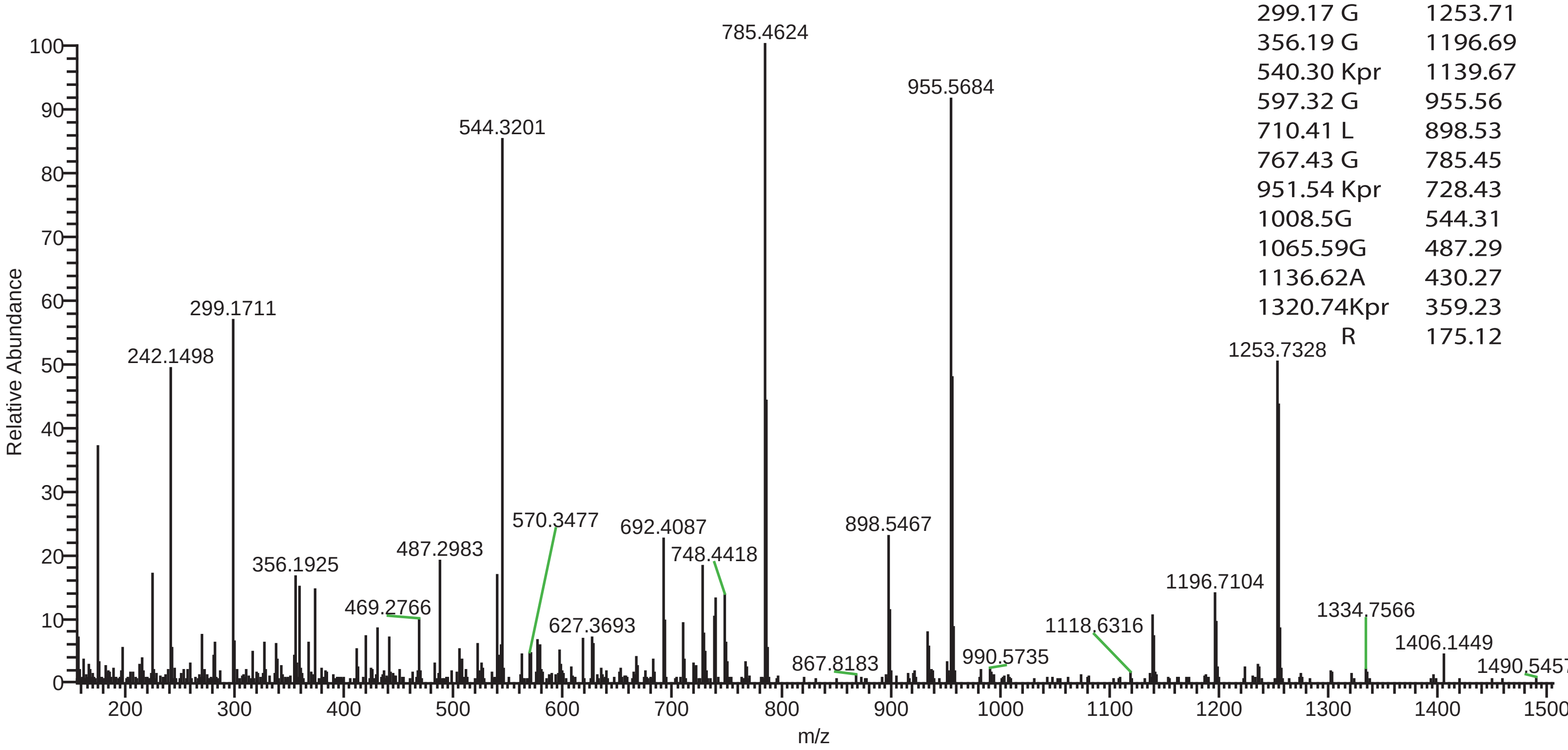

Peptide-6: H4K5ac/K8_12_16pr
GK(ac)GGK(pr)GLGK(pr)GGAK(pr)R

MS/MS 740.93^{2+}

b	Seq.	y
	G	1480.84
228.13	Kac	1423.82
285.16	G	1253.71
342.18	G	1196.69
526.29	Kpr	1139.67
583.31	G	955.56
696.40	L	898.53
753.42	G	785.45
937.53	Kpr	728.43
994.56	G	544.31
1051.58	G	487.29
1122.61	A	430.27
1306.73	Kpr	359.23
	R	175.12

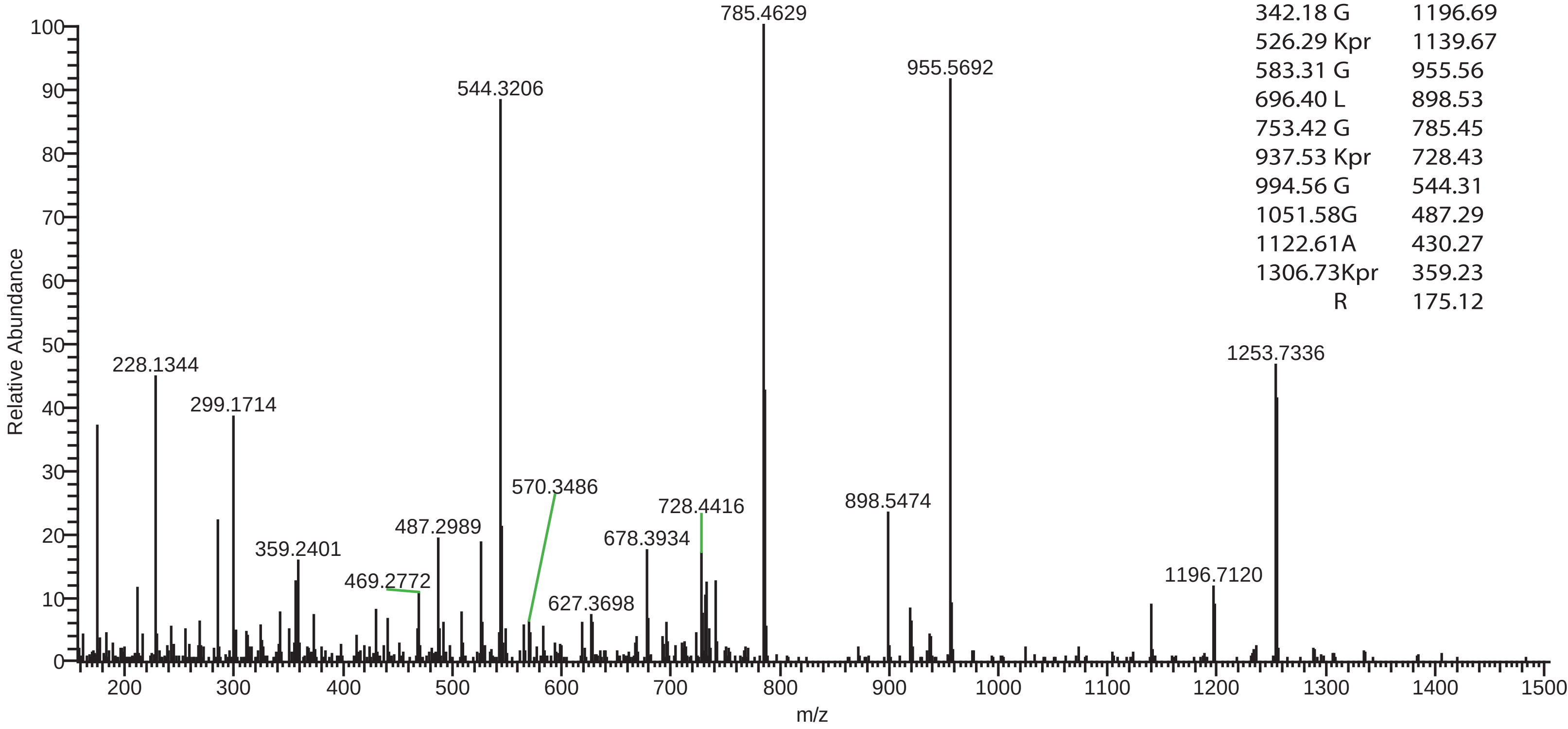

Peptide-7: H4K8ac/K5_12_16pr
GK(pr)GGK(ac)GLGK(pr)GGAK(pr)R

MS/MS 740.93^{2+}

b	Seq.	y
	G	1480.84
242.14	Kpr	1423.82
299.17	G	1239.70
356.19	G	1182.68
526.29	Kac	1125.66
583.31	G	955.56
696.40	L	898.53
753.42	G	785.45
937.53	Kpr	728.43
994.56	G	544.31
1051.58	G	487.29
1122.61	A	430.27
1306.73	Kpr	359.23
	R	175.12

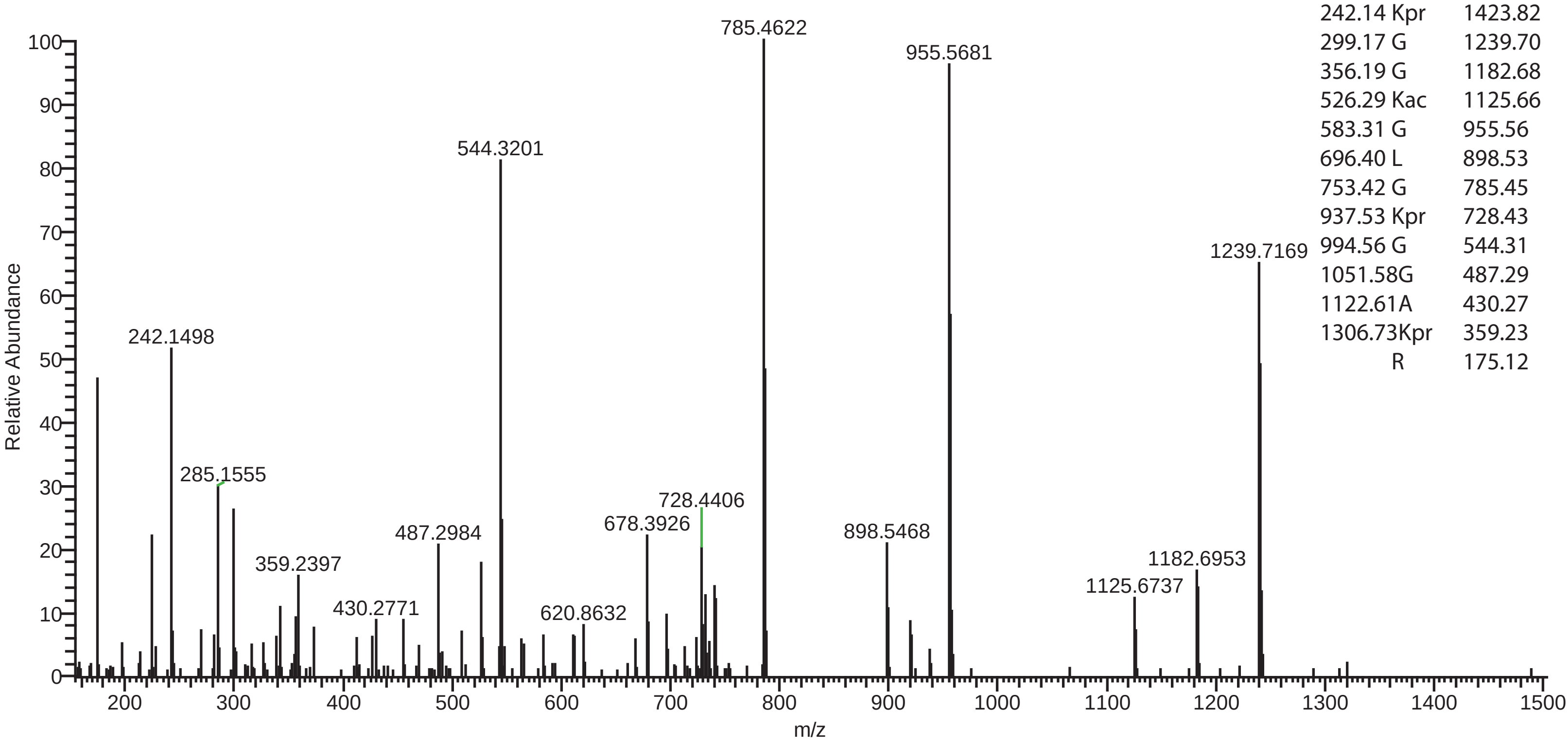

Peptide-8: H4K12ac/K5_8_16pr
GK(pr)GGK(pr)GLGK(ac)GGAK(pr)R

MS/MS 740.93^{2+}

b	Seq.	y
	G	1480.84
242.14	Kpr	1423.82
299.17	G	1239.70
356.19	G	1182.68
540.30	Kpr	1125.66
597.32	G	941.55
710.41	L	884.52
767.43	G	771.44
937.53	Kac	714.42
994.56	G	544.31
1051.58	G	487.29
1122.61	A	430.27
1306.73	Kpr	359.23
	R	175.12

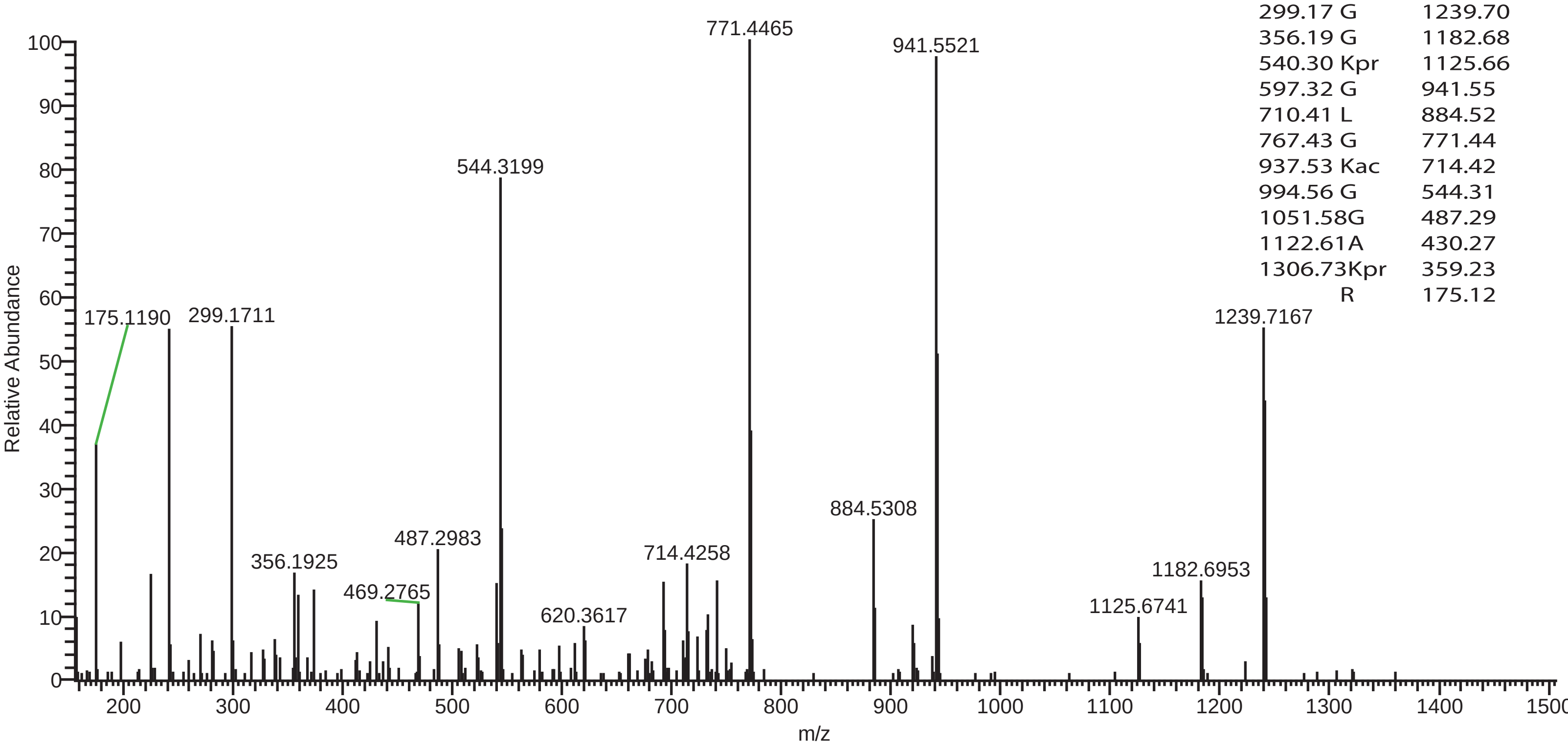

Peptide-9: H4K16ac/K5_8_12pr
GK(pr)GGK(pr)GLGK(pr)GGAK(ac)R

MS/MS 740.93^{2+}

b	Seq.	y
	G	1480.84
242.14	Kpr	1423.82
299.17	G	1239.70
356.19	G	1182.68
540.30	Kpr	1125.66
597.32	G	941.55
710.41	L	884.52
767.43	G	771.44
951.54	Kpr	714.42
1008.57	G	530.30
1065.59	G	473.28
1136.62	A	416.26
1306.73	Kac	345.22
	R	175.12

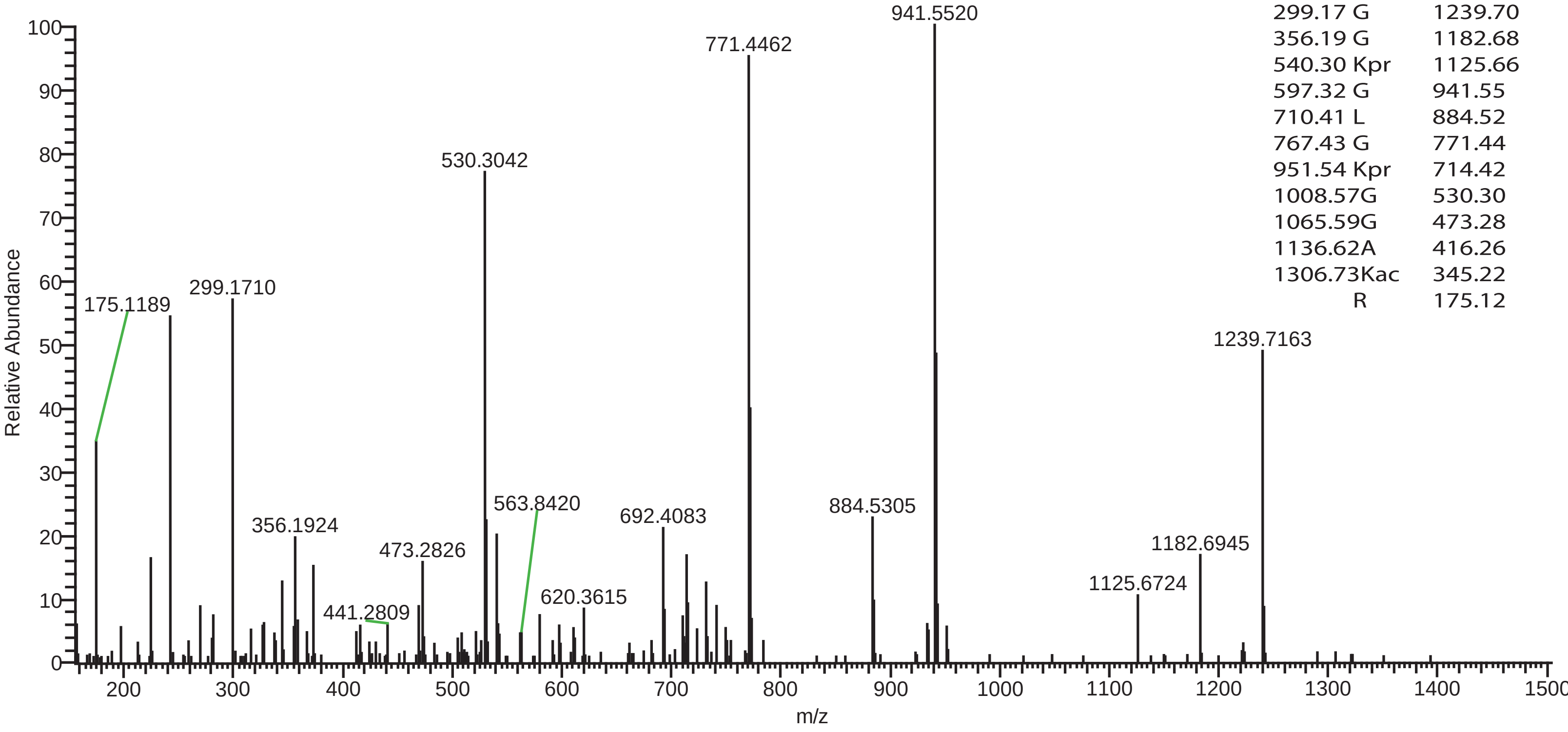

Peptide-10: H4K5_8ac/K12_16pr
GK(ac)GGK(ac)GLGK(pr)GGAK(pr)R

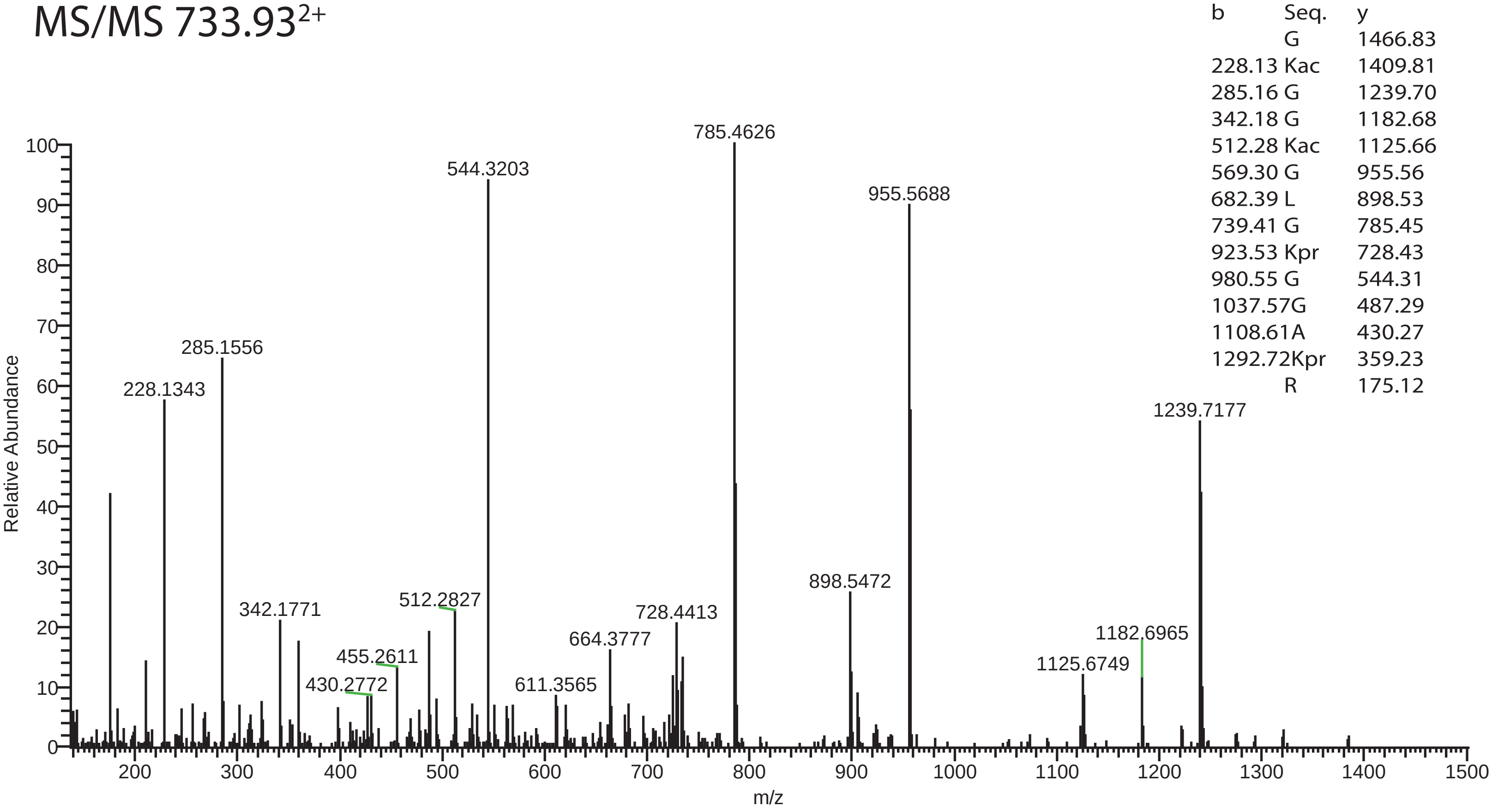

Peptide-11: H4K5_12ac/K8_16pr
GK(ac)GGK(pr)GLGK(ac)GGAK(pr)R

MS/MS 733.93^{2+}

b	Seq.	y
	G	1466.83
228.13	Kac	1409.81
285.16	G	1239.70
342.18	G	1182.68
526.29	Kpr	1125.66
583.31	G	941.55
696.40	L	884.52
753.42	G	771.44
923.53	Kac	714.42
980.55	G	544.31
1037.57	G	487.29
1108.61	A	430.27
1292.72	Kpr	359.23
	R	175.12

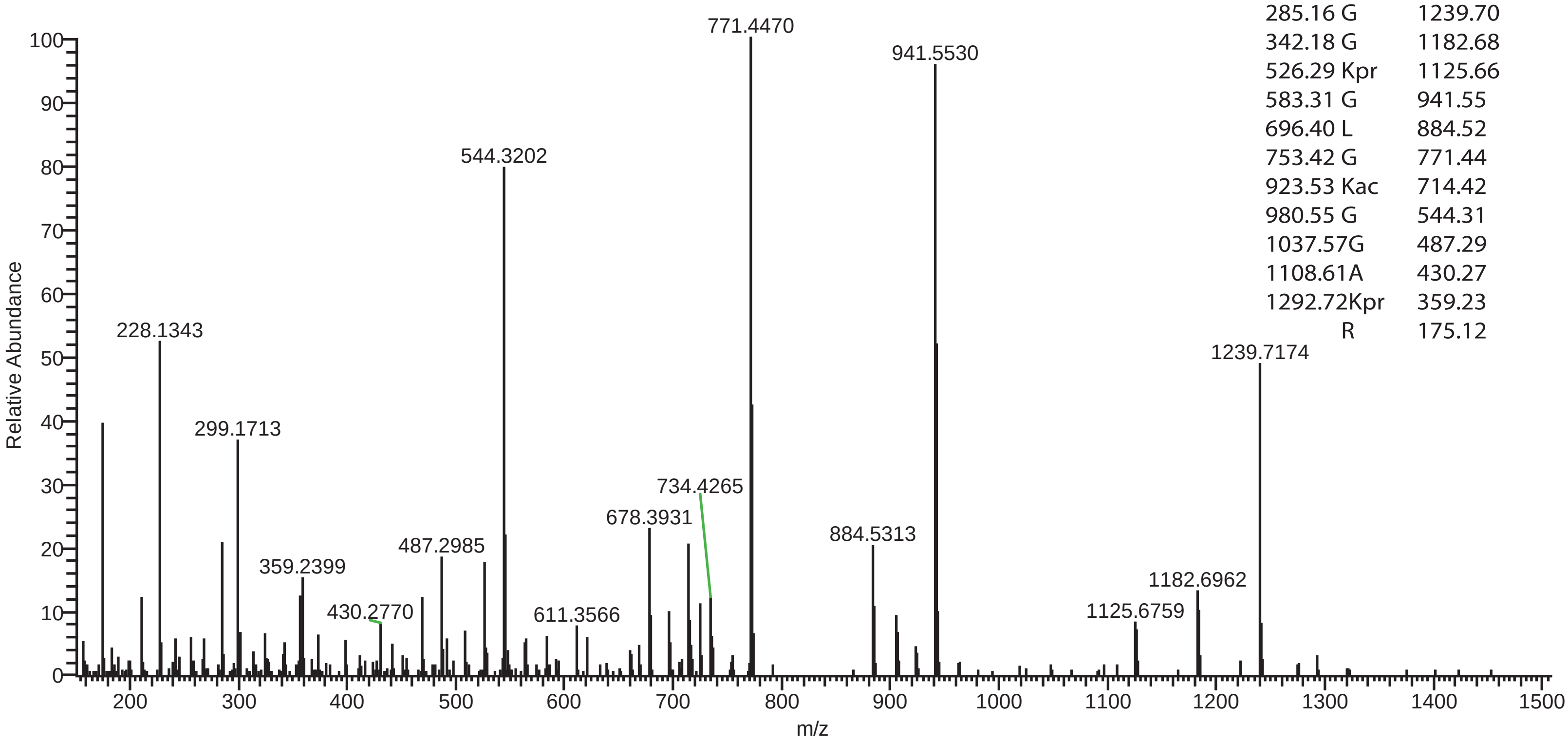

Peptide-12: H4K5_16ac/K8_12pr

GK(ac)GGK(pr)GLGK(pr)GGAK(ac)R

MS/MS 733.93^{2+}

b	Seq.	y
	G	1466.83
228.13	Kac	1409.81
285.16	G	1239.70
342.18	G	1182.68
526.29	Kpr	1125.66
583.31	G	941.55
696.40	L	884.52
753.42	G	771.44
937.53	Kpr	714.42
994.56	G	530.30
1051.58	G	473.28
1122.61	A	416.26
1292.72	Kac	345.22
	R	175.12

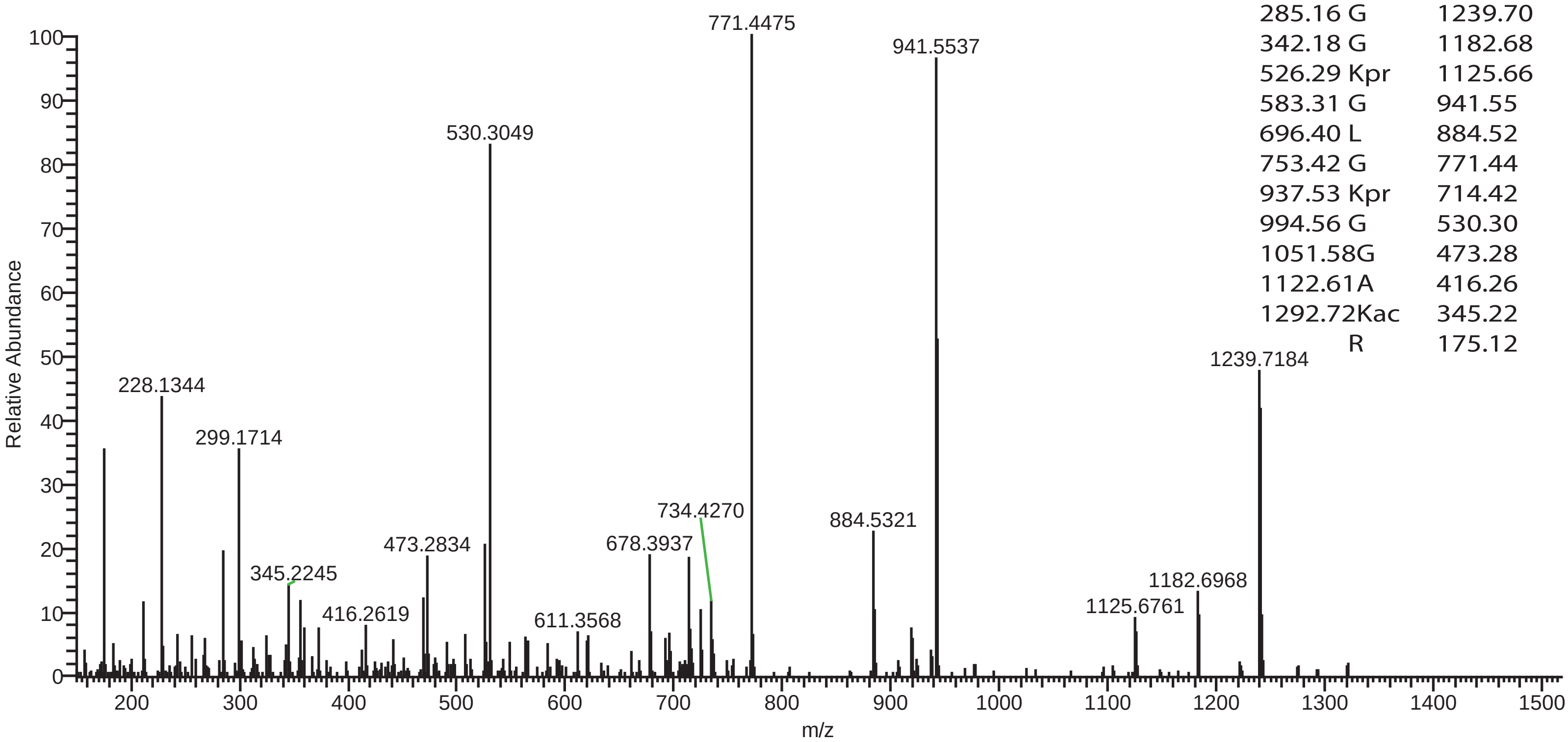

Peptide-13: H4K8_12ac/K5_16pr
GK(pr)GGK(ac)GLGK(ac)GGAK(pr)R

b	Seq.	y
	G	1466.83
242.14	Kpr	1409.81
299.17	G	1225.69
356.19	G	1168.67
526.29	Kac	1111.65
583.31	G	941.55
696.40	L	884.52
753.42	G	771.44
923.53	Kac	714.42
980.55	G	544.31
1037.57	G	487.29
1108.61	A	430.27
1292.72	Kpr	359.23
	R	175.12

MS/MS 733.93^{2+}

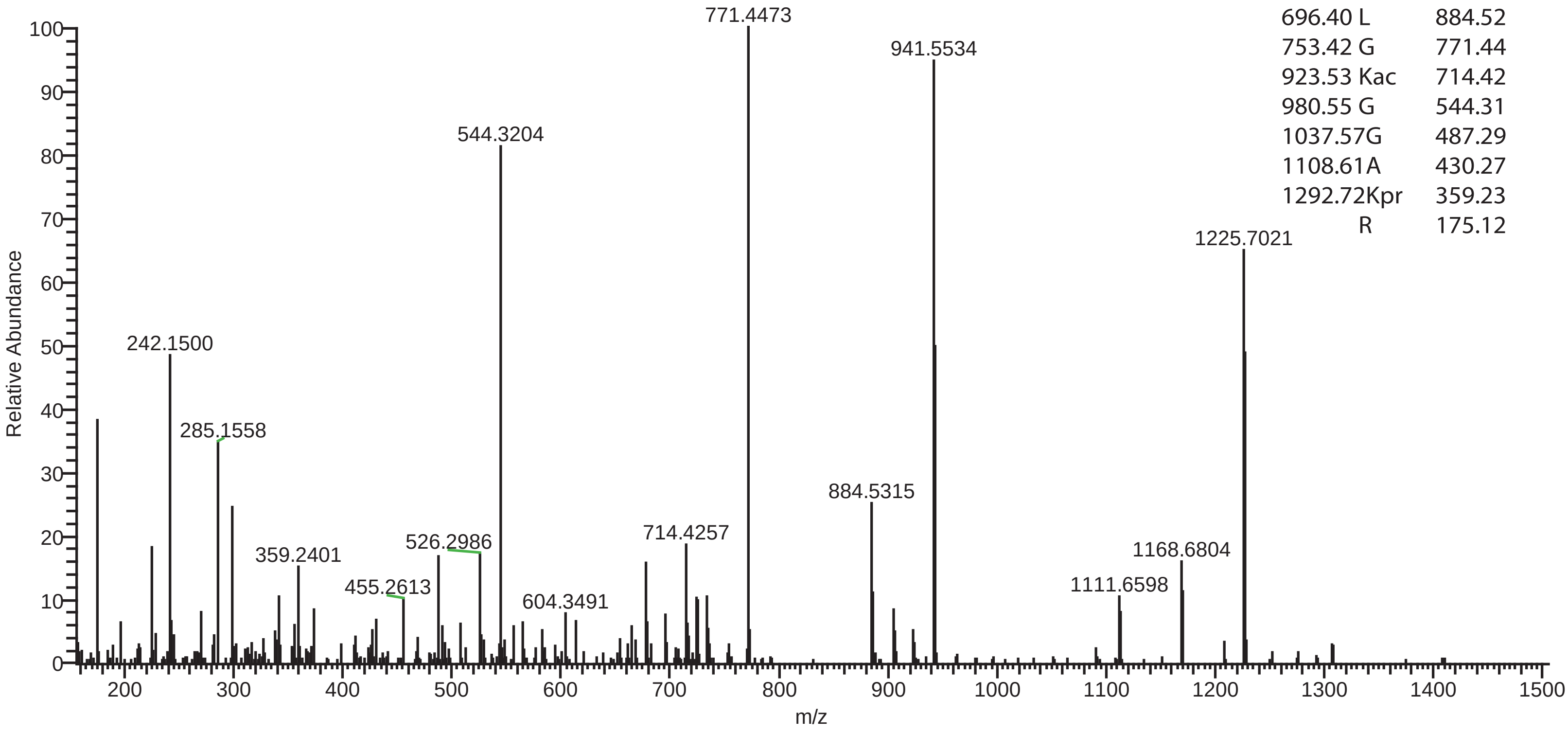

Peptide-14: H4K8_16ac/K5_12pr
GK(pr)GGK(ac)GLGK(pr)GGAK(ac)R

MS/MS 733.93^{2+}

b	Seq.	y
	G	1466.83
242.14	Kpr	1409.81
299.17	G	1225.69
356.19	G	1168.67
526.29	Kac	941.55
696.40	L	884.52
753.42	G	771.44
937.53	Kpr	714.42
994.56	G	530.30
1051.58	G	473.28
1122.61	A	416.26
1292.72	Kac	345.22
	R	175.12

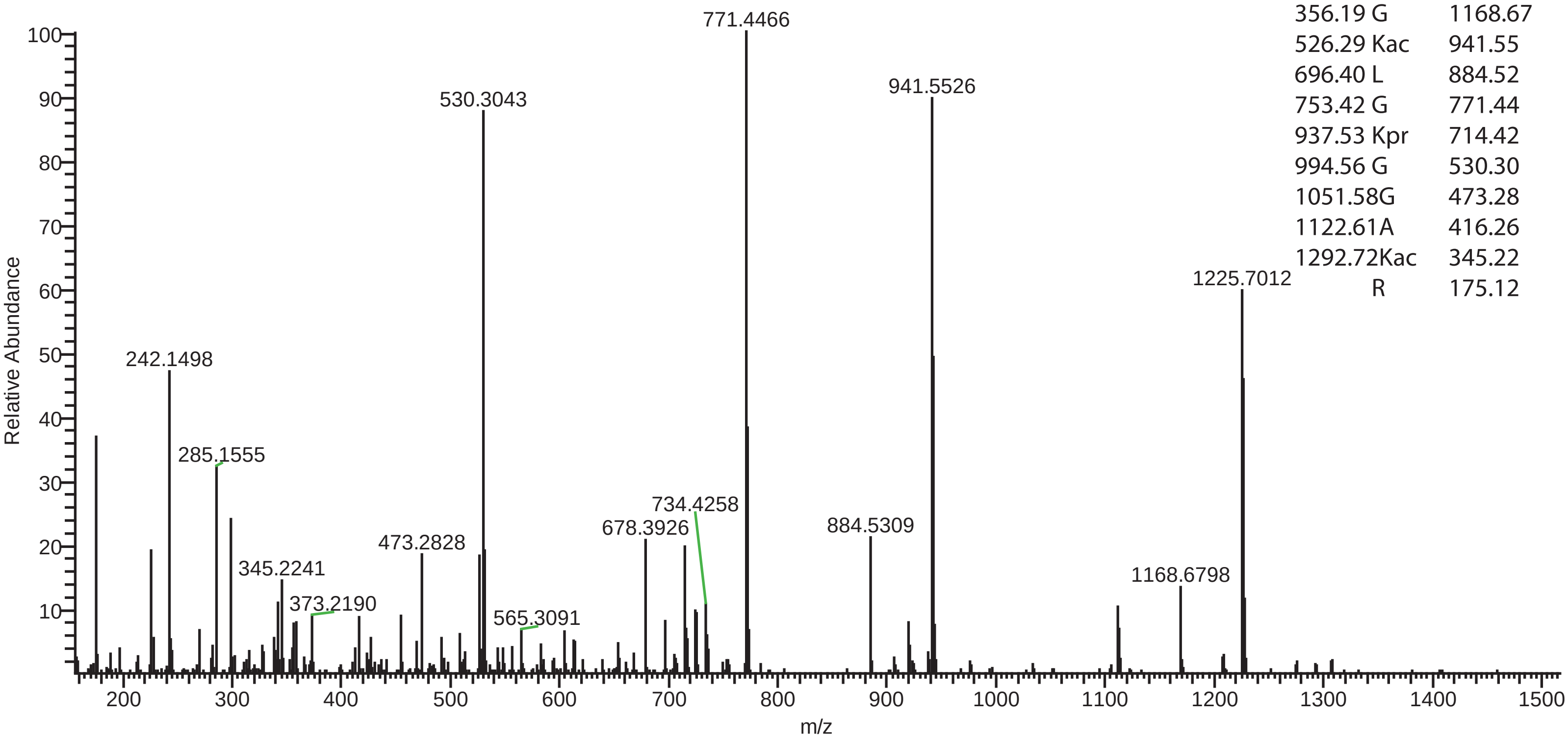

Peptide-15: H4K12_16ac/K5_8pr
GK(pr)GGK(pr)GLGK(ac)GGAK(ac)R

MS/MS 733.93^{2+}

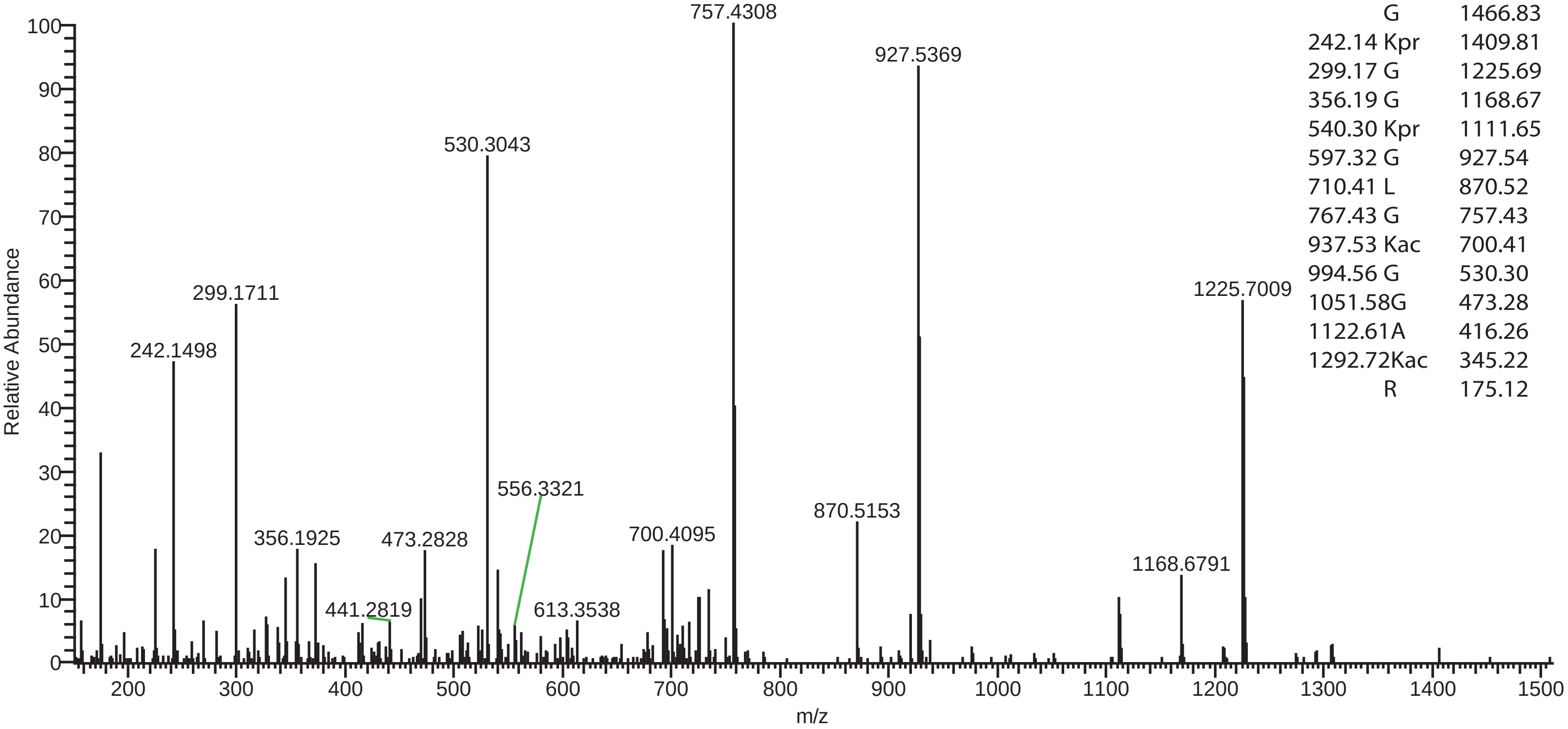

b	Seq.	y
	G	1466.83
242.14	Kpr	1409.81
299.17	G	1225.69
356.19	G	1168.67
540.30	Kpr	1111.65
597.32	G	927.54
710.41	L	870.52
767.43	G	757.43
937.53	Kac	700.41
994.56	G	530.30
1051.58	G	473.28
1122.61	A	416.26
1292.72	Kac	345.22
	R	175.12

Peptide-16: H4K5_8_12ac/K16pr
GK(ac)GGK(ac)GLGK(ac)GGAK(pr)R

MS/MS 726.92^{2+}

b	Seq.	y
	G	1452.82
228.13	Kac	1395.80
285.16	G	1225.69
342.18	G	1168.67
512.28	Kac	1111.65
569.30	G	941.55
682.39	L	884.52
739.41	G	771.44
909.52	Kac	714.42
966.54	G	544.31
1023.56	G	487.29
1094.60	A	430.27
1278.71	Kpr	359.23
	R	175.12

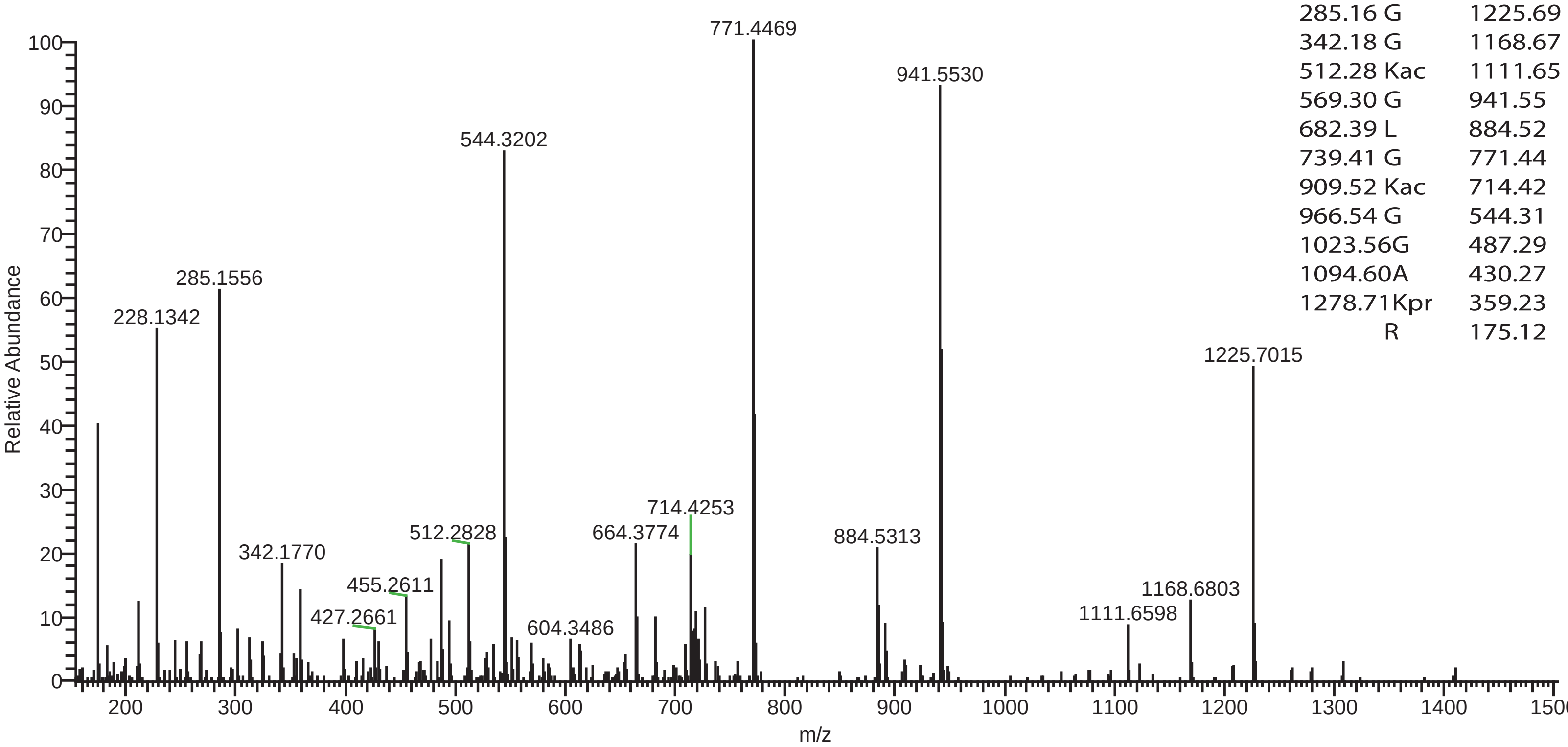

Peptide-17: H4K5_8_16ac/K12pr
GK(ac)GGK(ac)GLGK(pr)GGAK(ac)R

MS/MS 726.92^{2+}

b	Seq.	y
	G	1452.82
228.13	Kac	1395.80
285.16	G	1225.69
342.18	G	1168.67
512.28	Kac	1111.65
569.30	G	941.55
682.39	L	884.52
739.41	G	771.44
923.53	Kpr	714.42
980.55	G	530.30
1037.57	G	473.28
1108.61	A	416.26
1278.71	Kac	345.22
	R	175.12

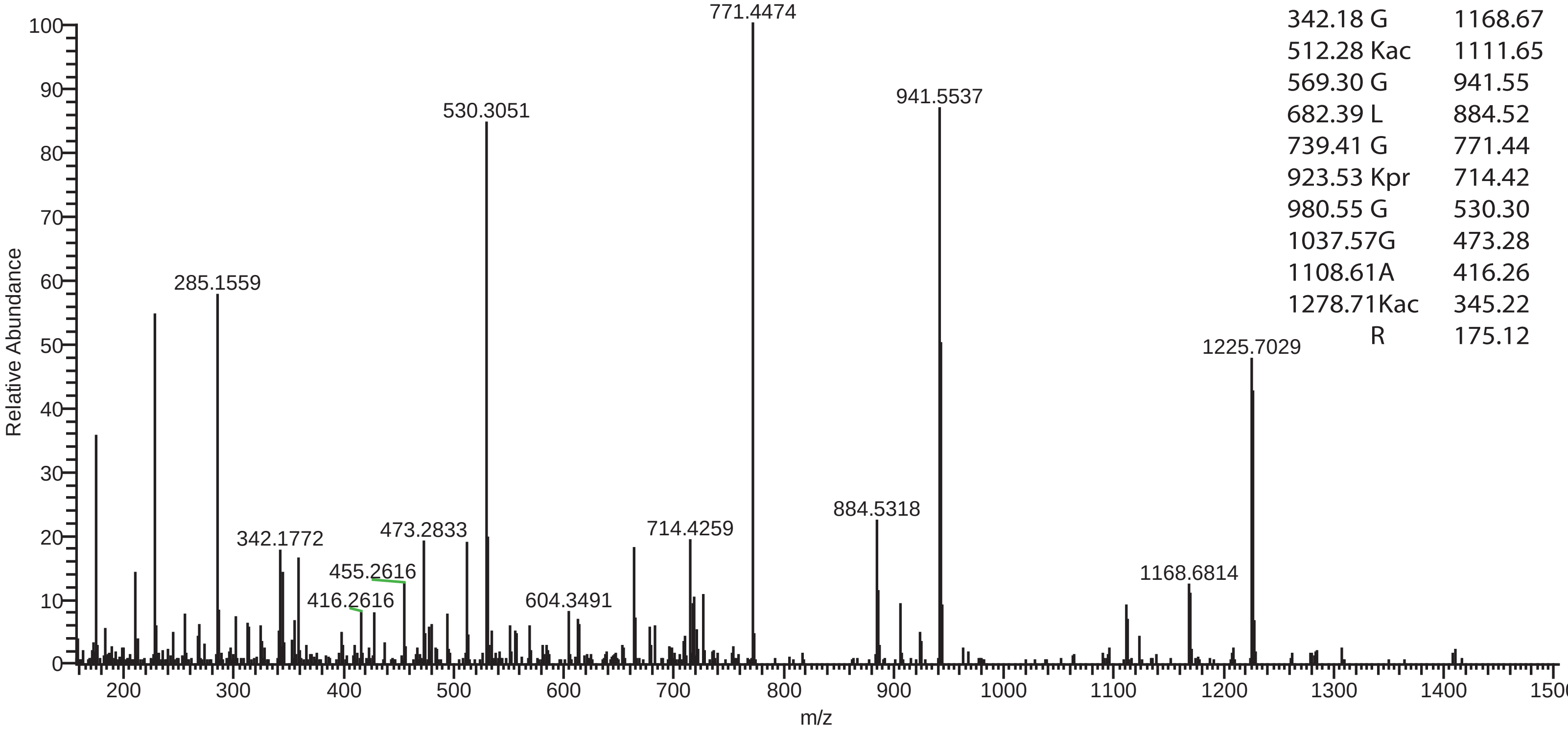

Peptide-18: H4K5_12_16ac/K8pr
GK(ac)GGK(pr)GLGK(ac)GGAK(ac)R

MS/MS 726.92^{2+}

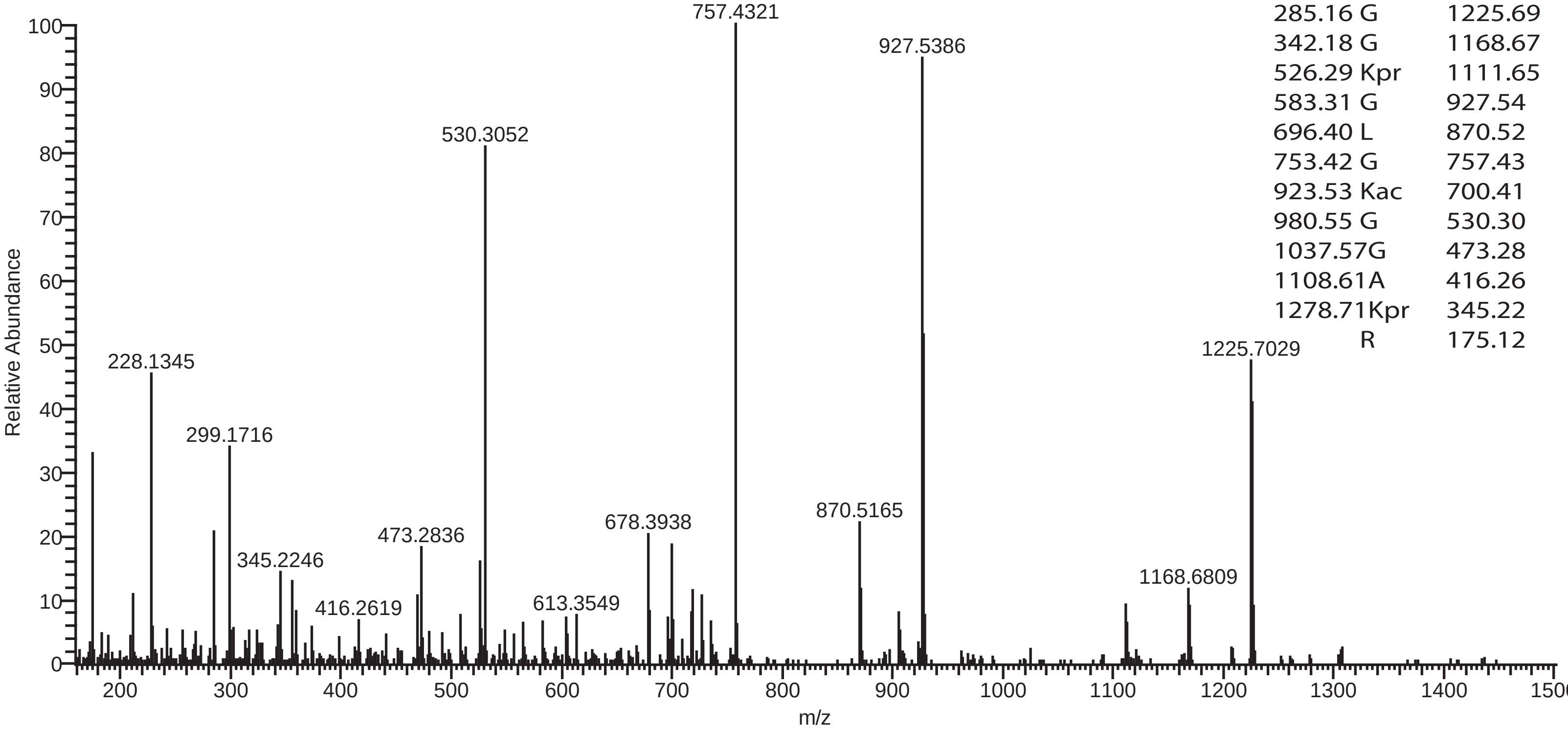

b	Seq.	y
	G	1452.82
228.13	Kac	1395.80
285.16	G	1225.69
342.18	G	1168.67
526.29	Kpr	1111.65
583.31	G	927.54
696.40	L	870.52
753.42	G	757.43
923.53	Kac	700.41
980.55	G	530.30
1037.57	G	473.28
1108.61	A	416.26
1278.71	Kpr	345.22
	R	175.12

Peptide-19: H4K8_12_16ac/K5pr
GK(pr)GGK(ac)GLGK(ac)GGAK(ac)R

MS/MS 726.92^{2+}

b	Seq.	y
	G	1452.82
242.14	Kpr	1395.80
299.17	G	1211.69
356.19	G	1154.66
526.29	Kac	1097.64
583.31	G	927.54
696.40	L	870.52
753.42	G	757.43
923.53	Kac	700.41
980.55	G	530.30
1037.57	G	473.28
1108.61	A	416.26
1278.71	Kac	345.22
	R	175.12

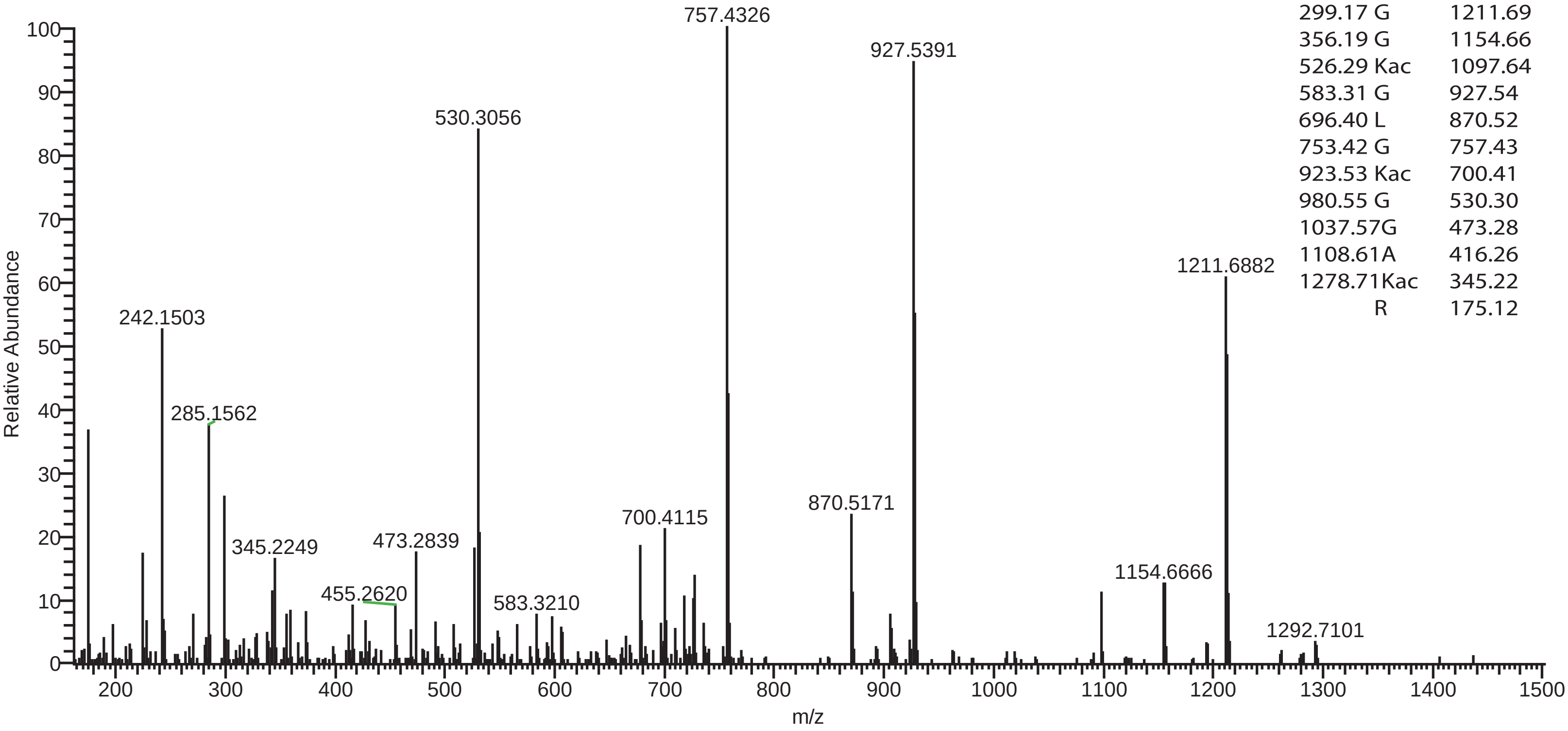

Peptide-20: H4K5_8_12_16ac
GK(ac)GGK(ac)GLGK(ac)GGAK(ac)R

MS/MS 719.91^{2+}

b	Seq.	y
	G	1438.81
228.13	Kac	1381.79
285.16	G	1211.69
342.18	G	1154.66
512.28	Kac	1097.64
569.30	G	927.54
682.39	L	870.52
739.41	G	757.43
909.52	Kac	700.41
966.54	G	530.30
1023.56	G	473.28
1094.60	A	416.26
1264.70	Kac	345.22
	R	175.12

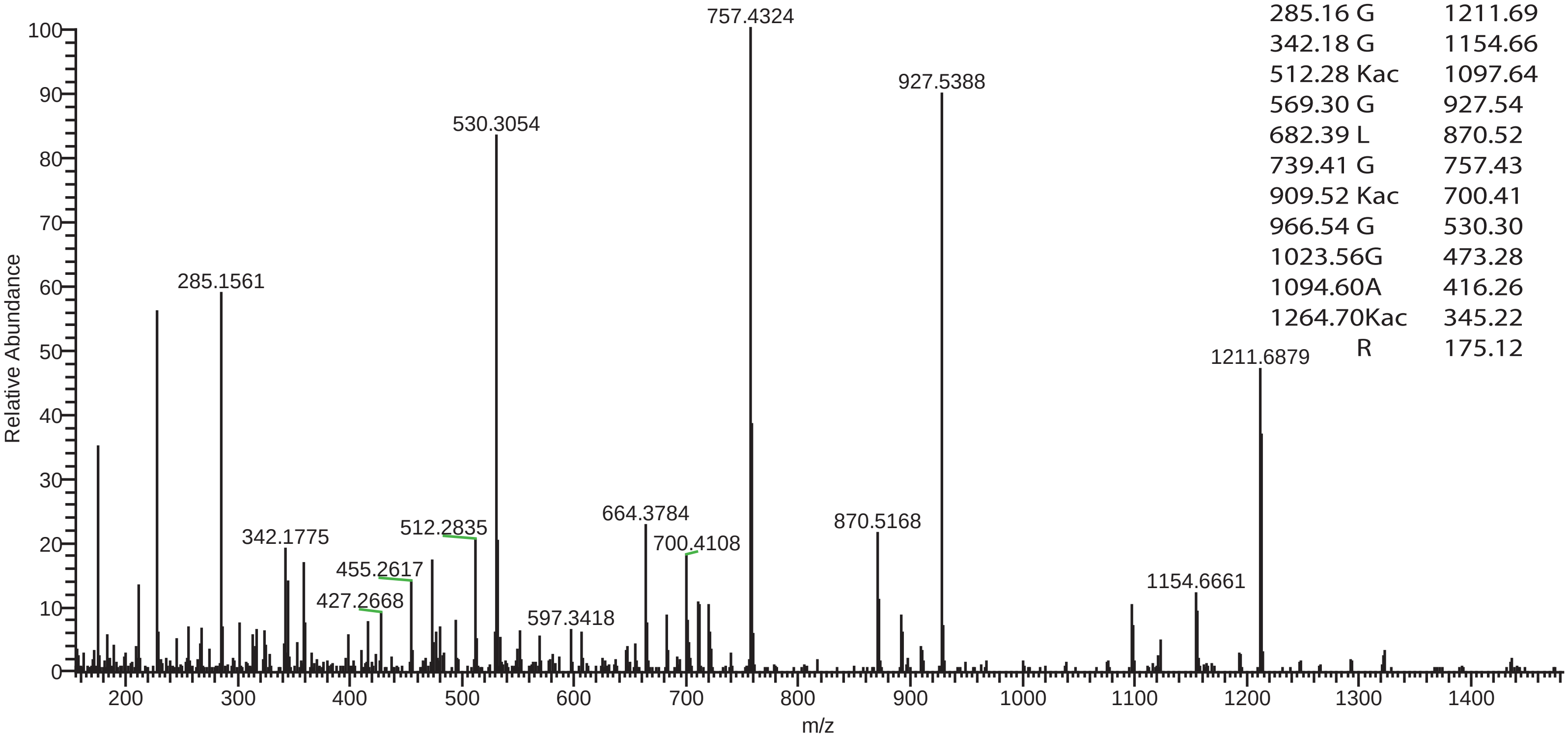

Supplementary Table 1: List of synthetic histone (A) H3 and (B) H4 peptides and their concentration in mM. The concentration of each peptide was determined based on triplicate UV absorbance measurements at 205 nm.

A)

H3 Peptides	Concentration (in mM)
Peptide 1: H3K18_K23NoAc	4.74
Peptide 2: H3K18Ac	5.58
Peptide 3: H3K23Ac	5.42
Peptide 4: H3K18_K23Ac	5.60

B)

H4 Peptides	Concentration (in mM)
Peptide 1: H4NoAc	0.60
Peptide 2: H4K5Ac	0.51
Peptide 3: H4K8Ac	0.57
Peptide 4: H4K12Ac	0.77
Peptide 5: H4K16Ac	0.79
Peptide 6: H4K5_8Ac	0.71
Peptide 7: H4K5_12Ac	0.68
Peptide 8: H4K5_16Ac	0.76
Peptide 9: H4K8_12Ac	0.70
Peptide 10: H4K8_16Ac	0.70
Peptide 11: H4K12_16Ac	0.71
Peptide 12: H4K5_8_12Ac	0.69
Peptide 13: H4K5_12_16Ac	0.75
Peptide 14: H4K8_12_16Ac	0.74
Peptide 15: H4K5_8_16Ac	0.82
Peptide 16: H4K5_8_12_16Ac	0.66

Supplementary Table 2: Synthetic H4 peptide standard dilutions. Standard curves for seven dilutions of mixtures of H4 (A) mono-, (B) di-, and (C) tri-acetylated peptides with peptide amounts ranging from 5 to 320 fmol were prepared. Two peptides from the mono- or tri-acetylated group and three peptides from the di-acetylated group were added to the mixture at fixed amount of 80 fmol.

A) Mono-acetylated isomers

	Peptide amount (fmol)			
Sample #	H4K5Ac	H4K8Ac	H4K12Ac	H4K16Ac
1	5	80	5	80
2	10	80	10	80
3	20	80	20	80
4	40	80	40	80
5	80	80	80	80
6	160	80	160	80
7	320	80	320	80

B) Tri-acetylated isomers

	Peptide amount (fmol)			
Sample #	H4K5_8_12Ac	H4K5_8_16Ac	H4K5_12_16Ac	H4K8_12_16Ac
1	5	80	5	80
2	10	80	10	80
3	20	80	20	80
4	40	80	40	80
5	80	80	80	80
6	160	80	160	80
7	320	80	320	80

C) Di-acetylated isomers

	Peptide amount (fmol)					
Sample #	H4K5_8Ac	H4K5_12Ac	H4K5_16Ac	H4K8_12Ac	H4K8_16Ac	H4K12_16Ac
1	5	80	5	80	5	80
2	10	80	10	80	10	80
3	20	80	20	80	20	80
4	40	80	40	80	40	80
5	80	80	80	80	80	80
6	160	80	160	80	160	80
7	320	80	320	80	320	80

Supplementary Table 3: Amount of each peptide isomer determined by Iso-PeptidAce after deconvolution of mixtures of known amounts of (A) four mono- (B) four tri- and (C) six di-acetylated isomeric groups. Sample at 80 fmols was used for normalisation of peptide intensities.

A) Mono-acetylated H4 peptides

Amount injected (fmol)	Amount determine by Iso-PeptidAce (fmol)			
	K5ac	K12ac	K8ac	K16ac
5	*1.33 ± 0.0	*5.05 ± 0.0	*74.28 ± 0.0	*74.52 ± 0.0
10	9.27 ± 4.4	12.36 ± 3.1	69.09 ± 7.51	66.59 ± 9.6
20	24.24 ± 8.2	25.17 ± 8.6	68.51 ± 8.9	67.18 ± 9.8
40	50.52 ± 12.3	49.49 ± 9.3	69.13 ± 8.7	68.23 ± 7.7
80	80	80	80	80
160	168.61 ± 4.4	181.21 ± 2.4	84.47 ± 7.6	86.99 ± 9.9
320	370.1 ± 58.4	401.15± 55.7	89.28 ± 0.3	89.21 ± 6.9

B) Tri-acetylated H4 peptides

Amount injected (fmol)	Amount determine by Iso-PeptidAce (fmol)			
	K5/8/12ac	K5/12/16ac	K5/8/16ac	K8/12/16ac
5	*1.92 ± 0.0	*2.44 ± 0.0	*65.4 ± 0.	*60.23 ± 0.0
10	7.47 ± 1.1	7.49 ± 0.3	93.9 ± 4.9	89.55 ± 10.1
20	15.38 ± 2.3	16.18 ± 1.2	99.13 ± 9.6	86.01 ± 7.6
40	35.1 ± 0.5	35.55 ± 1.0	95.25 ± 2.1	85.31 ± 9.7
80	80	80	80	80
160	155.82 ± 4.3	157.31 ± 8.7	76.56 ± 0.0	77.02 ± 1.5
320	295.55 ± 6.6	310.17 ± 20.9	78.86 ± 3.3	72.15 ± 3.4

C) Di-acetylated H4 peptides

Amount injected (fmol)	Amount determine by Iso-PeptidAce (fmol)					
	K5/8ac	K5/16ac	K8/16ac	K5/12ac	K8/12ac	K12/16ac
5	*3.7 ± 0.0	*1 ± 0.0	*6.92 ± 0	*70.98 ± 0.0	*286.89 ± 0	*114.68 ± 0.0
10	8.17 ± 1.0	4.31 ± 1.1	11.56 ± 2.0	85.64 ± 8.2	223.19 ± 8.7	110.13 ± 0.5
20	19.42 ± 4.8	17.03 ± 1.7	25.73 ± 1.8	96.52 ± 8.1	215.69 ± 5.2	114.52 ± 1.1
40	46.33 ± 2.9	52.58 ± 12.5	50.85 ± 4.1	101.69 ± 11.2	200.39 ± 26.2	115.94 ± 0.4
80	80	80	80	80	80	80
160	276.67 ± 21.2	459.33 ± 159.6	207.11 ± 3.9	104.26 ± 3.6	71.11 ± 3.6	98.01 ± 3.7
320	510.19 ± 63.7	1051.96 ± 500.8	372.77 ± 28.5	109.1 ± 0.6	65.78 ± 2.3	95.97 ± 1.37

Note: Each value represent the mean of two technical replicates + range error bar.

* This values were determined based on signal response of only one replicate

Supplementary Table 4 A-B : Calculated acetylation site occupancies of H3 and H4 peptides before or after HDACi treatment. Acetylation site occupancy of, for instance H4K5ac, was determined by dividing the sum of normalized intensities of all peptides containing acetylated Lys-5 by the sum of normalized intensities of all forms of acetylated and un-acetylated H4 peptide 4-GKGGKGLGKGGAKR-17.

A) H3 acetylation site occupancy

Sample	H3K18ac	H3K23ac
DMSO-1h	4.23 ± 0.14	26.56 ± 0.66
DMSO-6h	6.03 ± 0.14	31.45 ± 0.93
DMSO-24h	5.89 ± 0.74	32.06 ± 0.98
MS275-1h	5.74 ± 0.15	32.03 ± 4.85
MS275-6h	10.64 ± 1.89	51.95 ± 6.32
MS275-24h	27.11 ± 1.67	63.35 ± 0.81
SAHA-1h	17.19 ± 2.46	55.89 ± 0.89
SAHA-6h	31.94 ± 0.28	71.92 ± 1.22
SAHA-24h	37.70 ± 6.36	79.73 ± 0.77
JNJ-1h	15.58 ± 1.17	59.95 ± 0.02
JNJ-6h	32.76 ± 2.66	76.55 ± 1.39
JNJ-24h	44.17 ± 1.53	82.77 ± 1.21

B) H4 acetylation site occupancy

Sample	H4K5ac	H4K8ac	H4K12ac	H4K16ac
DMSO-1h	2.68 ± 0.59	2.63 ± 0.46	5.14 ± 0.53	29.27 ± 1.27
DMSO-6h	3.05 ± 0.07	2.86 ± 0.02	5.88 ± 0.04	28.42 ± 1.00
DMSO-24h	3.43 ± 0.60	3.79 ± 0.51	7.52 ± 0.98	32.27 ± 0.08
MS275-1h	3.42 ± 0.16	3.66 ± 0.35	7.12 ± 0.58	31.79 ± 0.52
MS275-6h	13.54 ± 0.78	13.43 ± 1.25	22.77 ± 1.21	57.81 ± 0.45
MS275-24h	36.79 ± 2.90	35.66 ± 1.47	55.16 ± 0.05	80.28 ± 0.23
SAHA-1h	16.24 ± 1.89	17.98 ± 1.65	33.03 ± 3.86	57.19 ± 0.79
SAHA-6h	44.96 ± 1.19	38.59 ± 1.91	63.45 ± 0.81	80.77 ± 0.17
SAHA-24h	61.35 ± 0.21	51.05 ± 3.30	77.49 ± 0.40	89.77 ± 1.16
JNJ-1h	13.51 ± 1.96	14.83 ± 1.43	26.48 ± 3.10	51.90 ± 3.30
JNJ-6h	47.15 ± 1.38	39.41 ± 0.06	64.59 ± 0.85	80.96 ± 0.49
JNJ-24h	57.67 ± 0.39	47.59 ± 1.18	68.25 ± 2.07	89.00 ± 0.80

Note: Each value represent the mean of two technical replicates ± range error bars.

Supplementary Table 5: LC/MS intensity data for CAF-1-bound and total histones H3 and H4 peptides.

H3 Peptide sequence	m/z	Charge	PTM	LC/MS Intensity	
				CAF-1 bound	Total histone
9-KSTGGKAPR-17	507.2906	2	H3K9prK14pr	860033.68 ± 231000	390383113.88 ± 11300000
	493.2748	2	H3K9acK14pr	255287.72 ± 17000	35576306.85 ± 12000000
	500.2836	2	H3K9prK14ac	415406.09 ± 2060	109502233.83 ± 2840000
		2	H3K9acK14ac	2253016.49 ± 418000	19487793.22 ± 5660000
18-KQLASKAAR-23	542.827	2	K18prK23pr	493253.48 ± 265000	703000000 ± 137000000
	535.819	2	K18acK23pr	28786.03 ± 26500	40300000 ± 14600000
		2	K18prK23ac	608652.18 ± 46000	149875000 ± 59300000
	528.811	2	K18acK23ac	58024.76 ± 34300	17545000 ± 6020000
27-KSAPSTGGVKKPHR-40	809.4568	2	K27prK36prK37pr	47760.38 ± 3200	46475000 ± 12900000
	816.4646	2	K27prK36pr+me1K37pr	0.00E+00	13272500 ± 4710000
	795.4595	2	K27prK36me2K37pr	0.00E+00	8742500 ± 300000
	530.642	3	K27prK36me2K37pr	0.00E+00	50900000 ± 2100000
	802.4673	2	K27prK36me3K37pr	0.00E+00	15470000 ± 600000
	535.3139	3	K27prK36me3K37pr	0.00E+00	80525000 ± 3000000
	802.4491	2	K27acK36prK37pr	43890.86 ± 6660	9195000 ± 300000
	809.4569	2	K27acK36pr+me1K37pr	0.00E+00	2970000 ± 800000
	788.4518	2	K27acK36me2K37pr	0.00E+00	574750 ± 100000
	525.9702	3	K27acK36me2K37pr	0.00E+00	2940000 ± 100000
	795.4596	2	K27acK36me3K37pr	0.00E+00	609250 ± 130000
	530.6421	3	K27acK36me3K37pr	0.00E+00	3507500 ± 1330000
	539.9736	3	K27prK36prK37pr	182908.65 ± 11300	168275000 ± 6040000
	544.6455	3	K27prK36pr+me1K37pr	0.00E+00	41650000 ± 15600000
	398.2333	4	K27prK36me2K37pr	0.00E+00	58900000 ± 21800000
	401.7373	4	K27prK36me3K37pr	0.00E+00	105650000 ± 38700000
	535.3018	3	K27acK36prK37pr	163800.96 ± 61100	29250000 ± 1380000
	539.9737	3	K27acK36pr+me1K37pr	0.00E+00	8445000 ± 1800000
	394.7295	4	K27acK36me2K37pr	0.00E+00	3520000 ± 1240000
	398.23334	4	K27acK36me3K37pr	0.00E+00	4467500 ± 1590000
54-FQK$_{56}$STELLIR-63	645.8743	2	K56pr	80480.47 ± 9580	47700000 ± 5940000
	638.8666	2	K56ac	197734.94 ± 39500	15050000 ± 1630000
53-RFQK$_{56}$STELLIR-63	723.9248	2	K56pr	23625.85 ± 20000	13207500 ± 2960000
	482.9523	3	K56pr	11242.59	11172500 ± 2580000
	478.2805	3	K56ac	49589.66	3972500 ± 1440000
	716.9171	2	K56ac	167034.28 ± 107000	4497500 ± 1250000

H4 Peptide sequence	m/z	Charge	PTM	LC/MS Intensity	
				CAF-1 bound	Total histone
4-GKGGKGLGKGGAKR-17	747.9412	2	K5-8-12-16pr	438525.03 ± 2090	21069549.87 ± 178000
	740.9334	2	K5ac\K8-12-16pr	0.00E+00	21208.89 ± 3630
			K8ac\K5-12-16pr	0.00E+00	357774.80 ± 39300
			K12ac\K5-8-16pr	1744.11 ± 2470	2410070.69 ± 145000
			K16ac\K5-8-12pr	24985.21 ± 35300	21740122.97 ± 1810000
	733.9255	2	K5-8ac\K12-16pr	0.00E+00	31596.76 ± 2540
			K5-12ac\K8-16pr	0.00E+00	347870.13 ± 25700
			K5-16ac\K8-12pr	0.00E+00	1666742.07 ± 389000
			K8-12ac\K5-16pr	0.00E+00	815051.26 ± 64900
			K8-16ac\K5-12pr	0.00E+00	2629819.07 ± 255000
			K12-16ac\K5-8pr	0.00E+00	8159580.12 ± 323000
	726.9177	2	K5-8-12ac\K16pr	488.08 ± 690	254396.05 ± 58200
			K5-8-16ac\K12pr	976.16	2092262.66 ± 58900
			K5-12-16ac\K8pr	110061.69 ± 18200	1909117.03 ± 582000
			K8-12-16ac\K5pr	17472.77 ± 12400	4395165.79 ± 1010000
	719.9092	2	K5-8-12-16ac	0.00E+00	3553895.37 ± 593000

Note: Each intensity value represents the mean of two technical replicates ± range error bar

Supplementary Table 6 : List of known acetylation sites in selected bromodomain substrates. Peptides containing multiple acetylated lysine residues generated from p53, GATA1, Cyclin T1, or MyoD are shown. List of publications for each of the previously identified acetylation sites are indicated.

Protein name	Interacting Bromodomain containing protein/s	Peptide segment containing multiple lysine residues	Known acetylation sites	Reference
p53	CREBBP, TAF1	290-RKKGEPHHELPPGSTKR-306	K292, K305	1-7
		306-RALPNNTSSSPQPKKKPLDGEYFTLQIR-333	K319, K320, K321	
		363- RAHSSHLKSKKGQSTSRHKKLMFKTEGP-390	K370, K372, K373, K381, K382, K386	
GATA1	BRD3	220- RTGHYLCNACGL YHKMNGQNRPLIRP KKR- 247	K233, K245, K246	8-10
		301- GIQTRNRKASGKGKKKRGSS -320	K308, K312, K314, K315, K316	
CyclinT1	BRD4	374-SQKQNSKSVPSAKVSLKEYR-393	K380, K386, K390	2, 11
MyoD	CREBBP or EP300	92- RCLLWACKACKRKTTNADR-110	K99, K102, K104	12, 13

1. Zeng, L., Zhang, Q., Gerona-Navarro, G., Moshkina, N. & Zhou, M.M. Structural Basis of Site-Specific Histone Recognition by the Bromodomains of Human Coactivators PCAF and CBP/p300. *Structure* 16, 643-652 (2008).
2. Choudhary, C. et al. Lysine Acetylation Targets Protein Complexes and Co-Regulates Major Cellular Functions. *Science* 325, 834-840 (2009).
3. Wu, S.Y. & Chiang, C.M. Crosstalk between sumoylation and acetylation regulates p53-dependent chromatin transcription and DNA binding, Vol. 28. (2009).
4. Joubel, A., Chalkley, R.J., Medzihradszky, K.F., Hondermarck, H. & Burlingame, A.L. Identification of New p53 Acetylation Sites in COS-1 Cells. *Molecular & Cellular Proteomics* 8, 1167-1173 (2009).
5. Sen, N., Kumari, R., Singh, Manika I. & Das, S. HDAC5, a Key Component in Temporal Regulation of p53-Mediated Transactivation in Response to Genotoxic Stress. *Molecular Cell* 52, 406-420 (2013).
6. Li, A.G. et al. An Acetylation Switch in p53 Mediates Holo-TFIID Recruitment. *Molecular Cell* 28, 408-421 (2007).
7. Tang, Y., Zhao, W., Chen, Y., Zhao, Y. & Gu, W. Acetylation Is Indispensable for p53 Activation. *Cell* 133, 612-626 (2008).
8. Boyes, J., Byfield, P., Nakatani, Y. & Ogryzko, V. Regulation of activity of the transcription factor GATA-1 by acetylation. *Nature* 396, 594-598 (1998).
9. Lamonica, J.M., Vakoc, C.R. & Blobel, G.A. Acetylation of GATA-1 is required for chromatin occupancy, Vol. 108. (2006).
10. Lamonica, J.M. et al. Bromodomain protein Brd3 associates with acetylated GATA1 to promote its chromatin occupancy at erythroid target genes. *Proceedings of the National Academy of Sciences* 108, E159-E168 (2011).
11. Schröder, S. et al. Two-pronged Binding with Bromodomain-containing Protein 4 Liberates Positive Transcription Elongation Factor b from Inactive Ribonucleoprotein Complexes. *Journal of Biological Chemistry* 287, 1090-1099 (2012).
12. Sartorelli, V. et al. Acetylation of MyoD Directed by PCAF Is Necessary for the Execution of the Muscle Program. *Molecular Cell* 4, 725-734 (1999).
13. Wei, L., Jamonnak, N., Choy, J., Wang, Z. & Zheng, W. Differential binding modes of the bromodomains of CREB-binding protein (CBP) and p300 with acetylated MyoD. *Biochemical and Biophysical Research Communications* 368, 279-284 (2008).

Supplementary Table 7. Representative raw intensity data obtained for deconvoluted H4 peptides for control and HDACi treated samples. For each sample Iso-PeptidAce reports five different groups of peptides: one un-acetylated (m/z 747.94), four mono-acetylated (m/z 740.93), six di-acetylated (m/z 733.93), four tri-acetylated (m/z 726.92) and one tetra-acetylated (m/z 719.91).

Sample	m/z	K5/8/12/16p	K5ac	K8ac	K12ac	K16ac	K5-8ac	K5-12ac	K5-16ac	K8-12ac	K8-16ac	K12-16ac	K5-8-12ac	K5-8-16ac	K5-12-16ac	K8-12-16ac	K5-8-12-16a
DMSO-1h	747.9412	53193490	0	0	0	0	0	0	0	0	0	0	0	0	0	0	0
DMSO-1h	740.9334	0	1118565.8	600295.66	1524585	20640811	0	0	0	0	0	0	0	0	0	0	0
DMSO-1h	733.9255	0	0	0	0	0	30356.163	121454.66	448997.57	164131.9	650668.81	1614132.7	0	0	0	0	0
DMSO-1h	726.9177	0	0	0	0	0	0	0	0	0	0	0	68875.319	196951.05	304594.31	464535.07	0
DMSO-1h	719.9099	0	0	0	0	0	0	0	0	0	0	0	0	0	0	0	228420.77
DMSO-6h	747.9412	330345784	0	0	0	0	0	0	0	0	0	0	0	0	0	0	0
DMSO-6h	740.9334	0	7138759.9	4371375.1	11037086	113532233	0	0	0	0	0	0	0	0	0	0	0
DMSO-6h	733.9255	0	0	0	0	0	106905.51	922259.1	2882531.3	1186154.7	4190846.2	10690293	0	0	0	0	0
DMSO-6h	726.9177	0	0	0	0	0	0	0	0	0	0	0	570076.9	685540.63	1500377.3	1935327.3	0
DMSO-6h	719.9099	0	0	0	0	0	0	0	0	0	0	0	0	0	0	0	956313.59
DMSO-24h	747.9412	252369980	0	0	0	0	0	0	0	0	0	0	0	0	0	0	0
DMSO-24h	740.9334	0	6805232.5	3776014.2	11013798	103256179	0	0	0	0	0	0	0	0	0	0	0
DMSO-24h	733.9255	0	0	0	0	0	144381.42	839325.07	2953775	1086588.8	4991609.9	13363139	0	0	0	0	0
DMSO-24h	726.9177	0	0	0	0	0	0	0	0	0	0	0	699796.5	1059935.7	1333050.3	3321485.9	0
DMSO-24h	719.9099	0	0	0	0	0	0	0	0	0	0	0	0	0	0	0	1922397.9
JNJ-1h	747.9412	117991108	0	0	0	0	0	0	0	0	0	0	0	0	0	0	0
JNJ-1h	740.9334	0	3579092.5	2065993.4	8538676.4	69116557	0	0	0	0	0	0	0	0	0	0	0
JNJ-1h	733.9255	0	0	0	0	0	95794.614	1799725.9	3389114.9	1299683.9	6819343.7	22637785	0	0	0	0	0
JNJ-1h	726.9177	0	0	0	0	0	0	0	0	0	0	0	869015.04	1471884	5756752.7	8917221.3	0
JNJ-1h	719.9099	0	0	0	0	0	0	0	0	0	0	0	0	0	0	0	15793648
JNJ-6h	747.9412	21343259	0	0	0	0	0	0	0	0	0	0	0	0	0	0	0
JNJ-6h	740.9334	0	1552352.3	912976.7	3398663.7	21578614	0	0	0	0	0	0	0	0	0	0	0
JNJ-6h	733.9255	0	0	0	0	0	0	3107565.6	3384182.8	679579.3	4960207.4	23855621	0	0	0	0	0
JNJ-6h	726.9177	0	0	0	0	0	0	0	0	0	0	0	353824.87	2558696.8	19685251	10314397	0
JNJ-6h	719.9099	0	0	0	0	0	0	0	0	0	0	0	0	0	0	0	44014522
JNJ-24h	747.9412	14354938	0	0	0	0	0	0	0	0	0	0	0	0	0	0	0
JNJ-24h	740.9334	0	4932701.3	1559004.9	4235243.7	31651823	0	0	0	0	0	0	0	0	0	0	0
JNJ-24h	733.9255	0	0	0	0	0	0	4836787.2	14322620	1958944.9	13580479	43687024	0	0	0	0	0
JNJ-24h	726.9177	0	0	0	0	0	0	0	0	0	0	0	1078741.7	15278200	44872091	21809930	0
JNJ-24h	719.9099	0	0	0	0	0	0	0	0	0	0	0	0	0	0	0	97722934
MS275-1h	747.9412	114207314	0	0	0	0	0	0	0	0	0	0	0	0	0	0	0
MS275-1h	740.9334	0	2785652.3	1408977.1	4263409.8	46273838	0	0	0	0	0	0	0	0	0	0	0
MS275-1h	733.9255	0	0	0	0	0	94043.304	403426.92	1186584.9	383662.27	2126216	4650092	0	0	0	0	0
MS275-1h	726.9177	0	0	0	0	0	0	0	0	0	0	0	224730.36	283902.84	547496.26	1181549.5	0
MS275-1h	719.9099	0	0	0	0	0	0	0	0	0	0	0	0	0	0	0	448649.1
MS275-6h	747.9412	78903759	0	0	0	0	0	0	0	0	0	0	0	0	0	0	0
MS275-6h	740.9334	0	6452819.9	2135734.5	7275288.4	66565880	0	0	0	0	0	0	0	0	0	0	0
MS275-6h	733.9255	0	0	0	0	0	42800.07	1926339.3	8568523.6	1232820.1	13482835	24691483	0	0	0	0	0
MS275-6h	726.9177	0	0	0	0	0	0	0	0	0	0	0	414625.37	3299476.3	6943465	7553312.1	0
MS275-6h	719.9099	0	0	0	0	0	0	0	0	0	0	0	0	0	0	0	5474857.5
MS275-24h	747.9412	56100816	0	0	0	0	0	0	0	0	0	0	0	0	0	0	0
MS275-24h	740.9334	0	13371602	3754090.1	15879233	109492331	0	0	0	0	0	0	0	0	0	0	0
MS275-24h	733.9255	0	0	0	0	0	403928.19	14301966	26611451	10427031	35744391	82346857	0	0	0	0	0
MS275-24h	726.9177	0	0	0	0	0	0	0	0	0	0	0	2461500.5	17916056	53442510	45254099	0
MS275-24h	719.9099	0	0	0	0	0	0	0	0	0	0	0	0	0	0	0	99427878
SAHA-1h	747.9412	75994058	0	0	0	0	0	0	0	0	0	0	0	0	0	0	0
SAHA-1h	740.9334	0	2592751.3	2160809.1	7380152.7	59218084	0	0	0	0	0	0	0	0	0	0	0
SAHA-1h	733.9255	0	0	0	0	0	36591.862	1948206.7	2900381.4	1263431.6	4426044.6	20909804	0	0	0	0	0
SAHA-1h	726.9177	0	0	0	0	0	0	0	0	0	0	0	919790.2	1030266.6	6108924.6	9786072.5	0
SAHA-1h	719.9099	0	0	0	0	0	0	0	0	0	0	0	0	0	0	0	16180294
SAHA-6h	747.9412	57211740	0	0	0	0	0	0	0	0	0	0	0	0	0	0	0
SAHA-6h	740.9334	0	4828466.5	1942512.3	9214826.5	68102682	0	0	0	0	0	0	0	0	0	0	0
SAHA-6h	733.9255	0	0	0	0	0	127801.71	10024666	10964147	2429316.3	16225382	67054445	0	0	0	0	0
SAHA-6h	726.9177	0	0	0	0	0	0	0	0	0	0	0	986651.9	7060824.5	54073678	28417899	0
SAHA-6h	719.9099	0	0	0	0	0	0	0	0	0	0	0	0	0	0	0	109799946
SAHA-24h	747.9412	15965728	0	0	0	0	0	0	0	0	0	0	0	0	0	0	0
SAHA-24h	740.9334	0	4526983.4	1489077.1	5341307.5	28573177	0	0	0	0	0	0	0	0	0	0	0
SAHA-24h	733.9255	0	0	0	0	0	224305.2	6828544.3	11230370	5217006.4	11394546	47756468	0	0	0	0	0
SAHA-24h	726.9177	0	0	0	0	0	0	0	0	0	0	0	3000516	12272353	59453258	32648488	0
SAHA-24h	719.9099	0	0	0	0	0	0	0	0	0	0	0	0	0	0	0	139482620

Supplementary Methods

Iso-PeptidAce : In-silico separation of co-eluting peptides

Overview

Here we describe Iso-PeptidAce, a stand-alone software for the in-silico separation of co-eluting peptide isomers in high resolution mass spectrometry data. Peptide isomers of interest need to be synthesized and processed individually in a high resolution mass spectrometer, with dynamic exclusion turned off. Samples with unknown proportions of co-eluting peptide isomers processed in the same instrument, with a similar method will be separated by the software. Iso-PeptidAce can also quantify (relatively) co-eluting peptides from mixed samples. To our knowledge, this is the first software capable of such a feat.

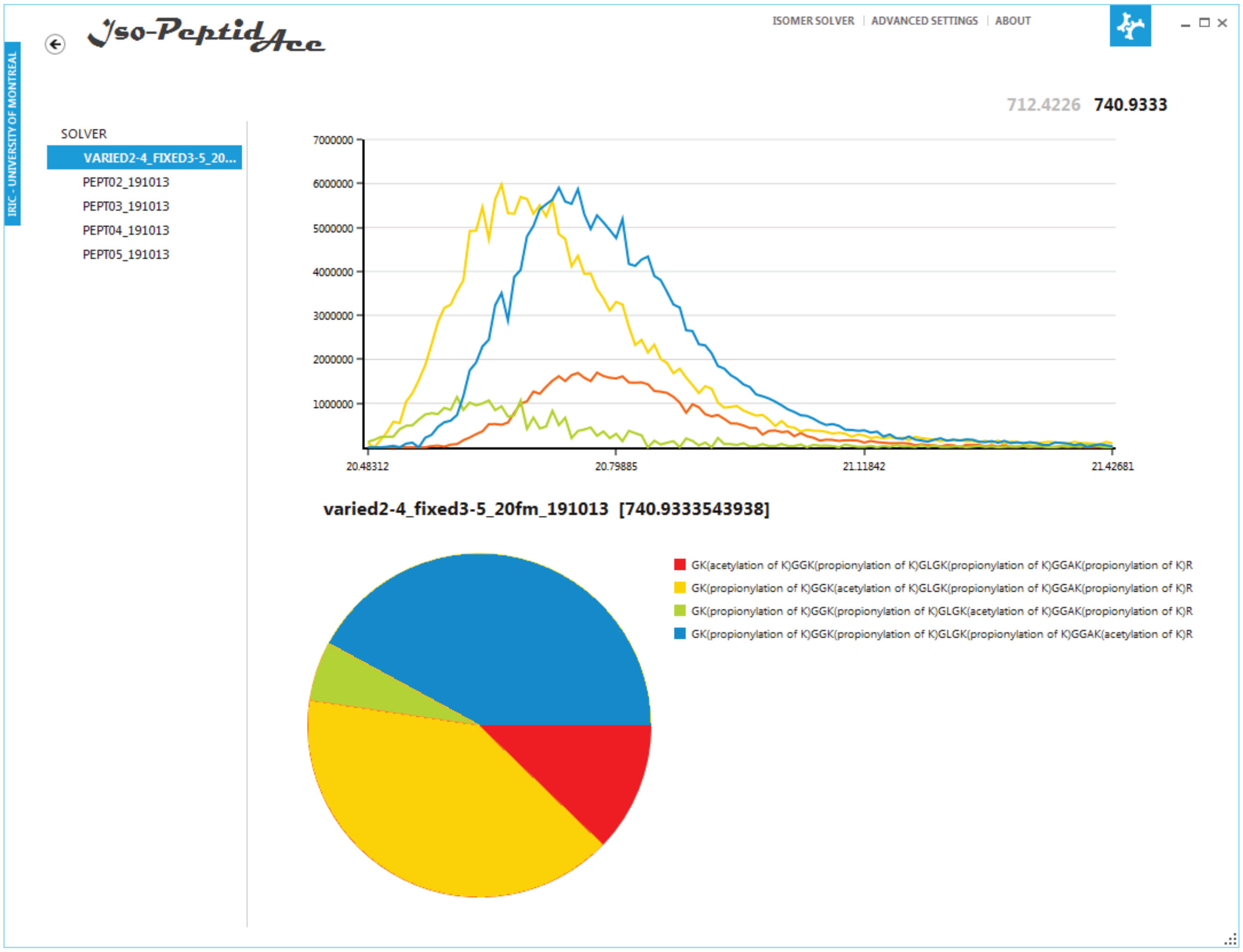

List of histone H4 peptides used for testing Iso-PeptidAce

Peptide 1	GK(propionylation of K)GGK(propionylation of K)GLGK(propionylation of K)GGAK(propionylation of K)R
Peptide 2	GK(acetylation of K)GGK(propionylation of K)GLGK(propionylation of K)GGAK(propionylation of K)R
Peptide 3	GK(propionylation of K)GGK(acetylation of K)GLGK(propionylation of K)GGAK(propionylation of K)R
Peptide 4	GK(propionylation of K)GGK(propionylation of K)GLGK(acetylation of K)GGAK(propionylation of K)R
Peptide 5	GK(propionylation of K)GGK(propionylation of K)GLGK(propionylation of K)GGAK(acetylation of K)R
Peptide 6	GK(acetylation of K)GGK(acetylation of K)GLGK(propionylation of K)GGAK(propionylation of K)R
Peptide 7	GK(acetylation of K)GGK(propionylation of K)GLGK(acetylation of K)GGAK(propionylation of K)R
Peptide 8	GK(acetylation of K)GGK(propionylation of K)GLGK(propionylation of K)GGAK(acetylation of K)R
Peptide 9	GK(propionylation of K)GGK(acetylation of K)GLGK(acetylation of K)GGAK(propionylation of K)R
Peptide 10	GK(propionylation of K)GGK(acetylation of K)GLGK(propionylation of K)GGAK(acetylation of K)R
Peptide 11	GK(propionylation of K)GGK(propionylation of K)GLGK(acetylation of K)GGAK(acetylation of K)R
Peptide 12	GK(acetylation of K)GGK(acetylation of K)GLGK(acetylation of K)GGAK(propionylation of K)R
Peptide 13	GK(acetylation of K)GGK(acetylation of K)GLGK(propionylation of K)GGAK(acetylation of K)R
Peptide 14	GK(acetylation of K)GGK(propionylation of K)GLGK(acetylation of K)GGAK(acetylation of K)R
Peptide 15	GK(propionylation of K)GGK(acetylation of K)GLGK(acetylation of K)GGAK(acetylation of K)R
Peptide 16	GK(acetylation of K)GGK(acetylation of K)GLGK(acetylation of K)GGAK(acetylation of K)R

Symbols used

- *MS1i, MS2i* = Injection Time for MS1 and MS2 Scans, in milliseconds
- *iMS* = ions per milliseconds
- *#ion* = Number of monoisotopic precursor ions fragmented in a scan
- *psm* = Peptide Spectrum Match
- *fi* = Fragment Intensity
- *nfi* = Normalized Fragment Intensity
- *n* = Number of fragments considered per characterized peptide
- *rt* = Scan Time
- *Δmz*= Precursor mass error, in ppm
- *mif* = Matching fragment ion divided by the number of theoretical ion tested
- c_j= Number of ions detected for peptide *j*
- *aMz* = List of selected fragment masses
- *ac* = Area Under the Curve
- *pc* = Peptide Count (Estimation of the number of a certain peptide in a sample)

Description of how spectral deconvolution is carried out by Iso-PeptidAce

Raw file extraction

Information is extracted from Raw files using ProteoWizard[1] and MsFileReader. MS2 peak lists (masses and intensities) are stored per precursor mass and sorted by retention time. Spectrum precursor intensity, MS1 and MS2 injection times are also extracted.

Precursor intensity counts are converted to ions per millisecond (*iMS*):

$$iMS = Precursor\ Intensity\ Count/MS1\ injection\ time$$

For each different precursor mass in a file, a curve describing the number of ions flowing in the system at any given time is built using the computed *iMS* values. Linear interpolation is used to compute the area under the curve for a given time point. In particular, the number of ions that entered the CTrap and got fragmented (*#ion*) is estimated as the area under the curve between the beginning (*rt*) and the end of the scan (*rt* + *MS2i*).

$$\#ion = Area\ Under\ The\ Curve\ (from{:}\ rt, to{:}\ rt + MS2i)$$

These operations are made for each file (both mixed samples and synthetic peptide runs). For the synthetic peptide runs, spectrum fragment intensity counts (*fi*) are normalized (*nfi*) using the number of ions (*#ion*) that got fragmented.

$$nfi = fi/\#ion$$

Peptide Spectrum Matching

Iso-PeptidAce uses PeptidAce peptide spectrum matching abilities (which derives from Morpheus) to automatically identify peptide sequences and modifications across synthetic peptide runs. PeptidAce uses a no-enzyme in-silico protein digestion routine to parse the provided Fasta file for potential matches. All modifications (specified in the "Modifications.csv" user configurable Comma Separated Values file) are searched for. This automatic identification is useful to discover impurities in the peptide synthesis process.

PeptidAce matches peptides to spectrum by comparing theoretical ions (fragments *a*, *b*, *c*, *x*, *y* and *z*) within a specified ppm tolerance window. Peptide spectrum matches (*psm*) are scored by their cumulated normalized fragment intensities (*nfi*), number of matching ions versus unmatched theoretical ions (*mif*) and precursor accuracy (Δmz, normalized between [0,1]):

$$psm\ score = 0.33 \cdot \Delta mz + 0.33 \cdot mif + 0.33 \cdot nfi$$

In case of discrepancy (precursor with inconsistent *psm* across the elution curve), *psm* scores of similar peptides are summed together and the best peptide is associated to the precursor. Ambiguous spectra assigned to different peptides are discarded.

Describing the system as a Maximum Flow problem

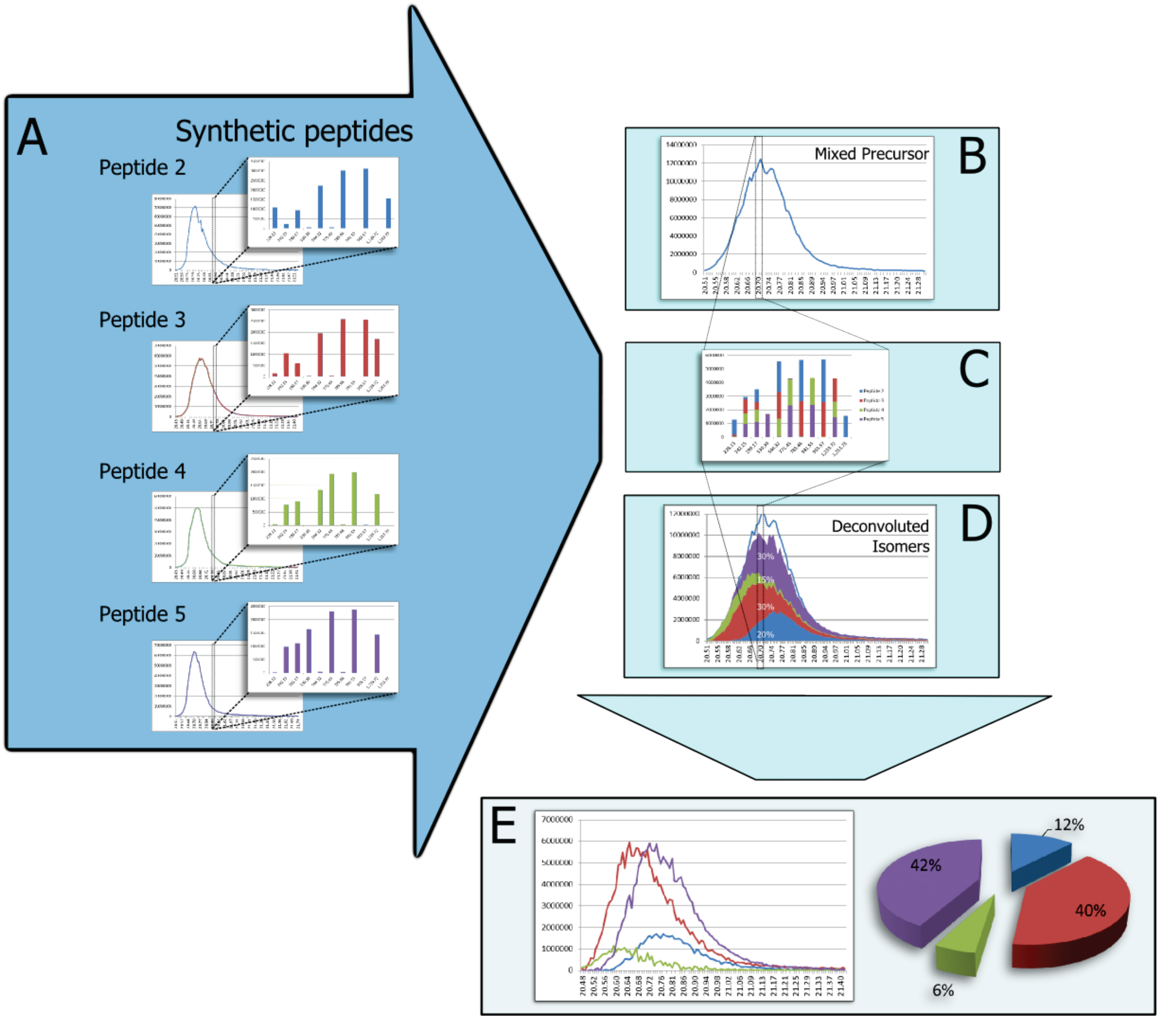

The network flow was built from mixed spectra and spectra acquired from peptide isomers. The objective, finding the maximum flow of the system, is stated as such:

How many ions of peptides isomer does it take to optimally fill the mixed spectrum fragment intensity counts

For each synthetic peptide found, normalized fragment intensities (*nfi*) of every spectrum were averaged. The *n* most intense fragments were chosen for each synthetic peptide. Only fragment masses with consistent normalized intensities (standard deviation above 50% of the mean) were

considered reproducible enough. Unstable fragments were flagged and ignored in following analyses.

For synthetic peptides of similar masses, these n fragments were used to build a list of fragment masses (aMz), both with common and unique fragment masses. The *nfi* of their averaged spectrum was used to associate intensities to a list of fragment masses (aMz_j, one aMz for each potential isomer *j*). A similar list of intensity count (aMz_m) is built for the mixed spectrum m of matching precursor mass. For each mixed spectrum m, the Maximum Flow is defined by the sum of aMz_j that optimally fills the aMz_m fragment intensity counts (were c_j is the number of ions for the *j* isomer, and aMz_t is the theoretical list being built):

$$aMz_t = \sum_{isomers}^{j}(c_j \cdot aMz_j) \text{ and } aMz_t \leq aMz_m$$

Thus, the network flow can be described as the source being connected to peptide isomers *j* (edges of unknown capacity) which are themselves connected to every selected fragments *aMz* (edge capacity determined by c_j) linking to the sink (edge capacity determined by aMz_j). When the maximum flow is reached, the first set of edges from the source represents the ratio of each peptide isomer found in the mixed spectrum.

Gradient Descent

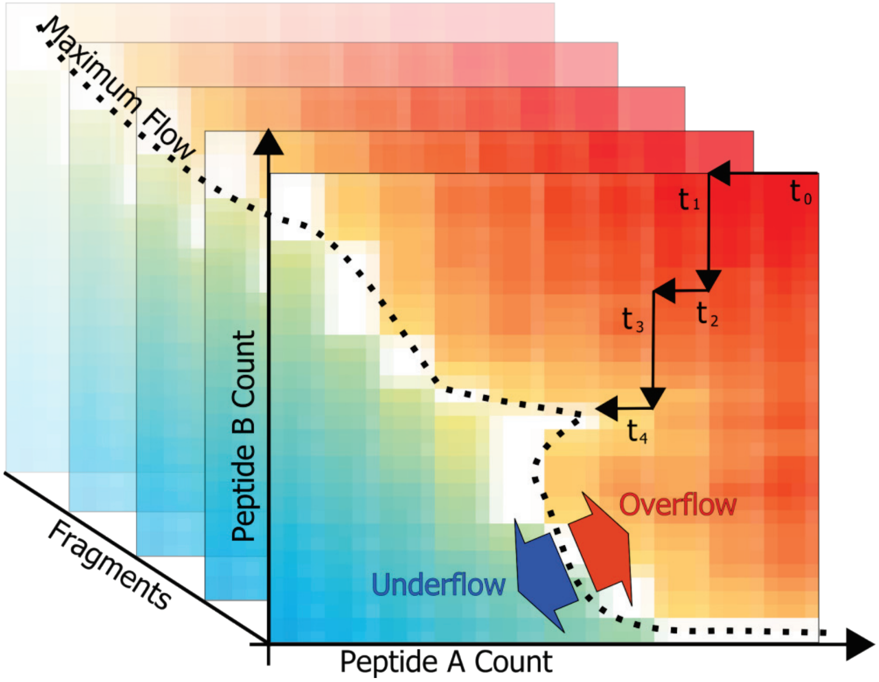

Because of the dependencies between fragment intensities within a synthetic peptide spectrum, traditional approaches (such as PreFlow Push) could not be used. Instead, a Gradient Descent was implemented.
The Gradient Descent is an iterative approach with each step being closer to the Maximum Flow than the previous iteration. The initial state of the gradient descent (c value for each j isomer) is computed as the number of times each j isomer can fit in the mixed spectrum.

$$c_j = aMz_m / aMz_j$$

This puts the system in an Overflow state (some fragment intensities within the theoretical aMz (aMz_t) are over their capacity). There might also be Underflow (fragments of aMz_t lower than their capacity in aMz_m).

Iteratively, until there is no Overflow in the network, the j isomer with the highest Overflow/Underflow score (objective function) has its c_j value reduced (ions are removed one unit at a time). Ambiguous states are resolved randomly.

Deconvolution

In PeptidAce, the peak tops and areas under the curve of precursors are both reported for quantification purposes. Iso-PeptidAce also reports area under the curve of the individual isomers populating a mixed precursor elution profile. For each isomer j, the number of ions populating the spectrum (deduced by each Gradient Descent result) is used to compute the number of ions per millisecond flowing in the system at different time points. The resulting, deconvoluted curve is traced through linear interpolation, which is in turn used to compute the area under the curve (ac_j) for that peptide.

Quantification

If peptide intensities are properly normalized based on the synthetic sample runs, it is possible to use this method for absolute quantification of peptides. When concentrations of spiked synthetic peptides are known, Iso-PeptidAce's area under the curve can be converted into absolute peptide counts. Assuming that synthetic peptide samples were processed exactly as the mixed spectrum samples, the number of peptides j in the sample m (pc_{mj}) is defined by the area under the curve (ac_{mj}) normalized by the synthetic peptide sample s information (peptide count in sample pc_{sj}, and area under the curve ac_{sj}).

$$pc_{mj} = ac_{mj} \cdot \left(pc_{sj} / ac_{sj}\right)$$

Results

Experimental design

To assess the precision of the approach, we designed an experiment to test deconvolution across different peptide isomer concentration ratios. Because of its high level of complexity, characterization of acetylation sites in the Histone H4 peptide **[1]** was selected. The software method was tested for the mono, di and tri-acetylated versions of the peptides while all other lysine sites were propyonylated. The next table depicts the concentrations normalized using the equimolar run (observed / expected):

	5fMol - 80fMol	10fMol - 80fMol	20fMol - 80fMol	40fMol - 80fMol	80fMol - 80fMol	160fMol - 80fMol	320fMol - 80fMol
Peptide 2	0.0 / 5	7.4 / 10	19.8 / 20	41.9 / 40	80.0 / 80	168.3 / 160	329.8 / 320
Peptide 3	72.7 / 80	74.0 / 80	72.9 / 80	73.6 / 80	80.0 / 80	74.8 / 80	83.7 / 80
Peptide 4	8.2 / 5	13.0 / 10	19.0 / 20	45.4 / 40	80.0 / 80	189.6 / 160	362.0 / 320
Peptide 5	75.7 / 80	75.9 / 80	78.1 / 80	74.8 / 80	80.0 / 80	72.9 / 80	81.9 / 80
Peptide 6	2.8 / 5	6.3 / 10	14.6 / 20	30.5 / 40	80.0 / 80	168.6 / 160	319.7 / 320
Peptide 7	53.0 / 80	60.6 / 80	67.8 / 80	69.6 / 80	80.0 / 80	74.1 / 80	82.2 / 80
Peptide 8	0.0 / 5	0.4 / 10	7.8 / 20	32.0 / 40	80.0 / 80	382.8 / 160	884.9 / 320
Peptide 9	215.8 / 80	177.4 / 80	150.4 / 80	165.4 / 80	80.0 / 80	44.4 / 80	27.1 / 80
Peptide 10	5.4 / 5	11.7 / 10	21.8 / 20	36.6 / 40	80.0 / 80	133.4 / 160	228.8 / 320
Peptide 11	78.5 / 80	81.9 / 80	82.8 / 80	84.6 / 80	80.0 / 80	66.6 / 80	66.4 / 80
Peptide 12	3.0 / 5	7.5 / 10	17.9 / 20	35.3 / 40	80.0 / 80	162.2 / 160	303.5 / 320
Peptide 13	93.8 / 80	92.4 / 80	90.4 / 80	95.1 / 80	80.0 / 80	67.8 / 80	71.6 / 80
Peptide 14	7.0 / 5	11.1 / 10	21.9 / 20	38.8 / 40	80.0 / 80	162.8 / 160	332.8 / 320
Peptide 15	79.5 / 80	81.8 / 80	81.0 / 80	78.8 / 80	80.0 / 80	73.7 / 80	65.2 / 80

Automatic identification of synthetic peptides

Automatic identification of peptide sequence and modification did not yield in any particular error. However, we did find impurities in the synthesized peptide samples. For instance, incomplete or non-acetylated forms of the peptides were seen in small quantities (less than 0.2%

of total intensity). Cross contamination of peptide forms was tested by running Iso-PeptidAce on sample containing only one peptide form. The resulting contamination intensity detected were too low to be worth accounting for.

Consistency of the Gradient Descent approach

Despite the fact that each gradient descent is done independently of the others, the results are surprisingly consistent across the mixed elution profile. The deconvoluted elution profiles presented in this next figures are comparable to the profiles drawn from the synthetic peptide runs with conserved elution time order and consistent intensity ratios across the profile (no bulge).

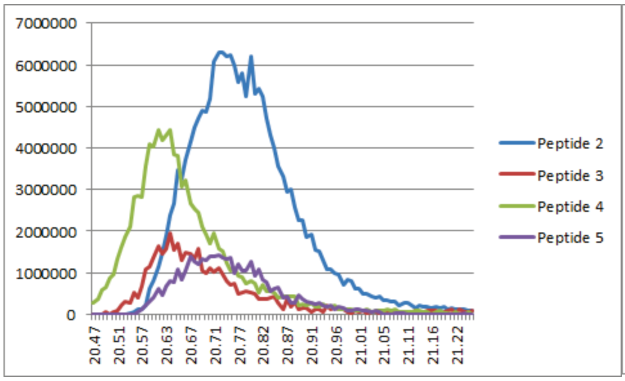

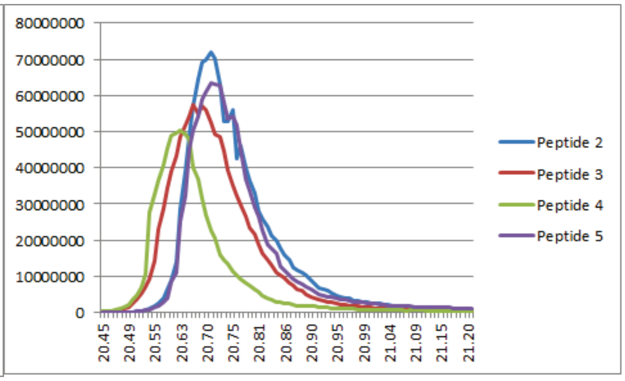

Expected versus Observed Concentration

The precision of the method is shown in the next figure, where observed peptide concentration ratios are plotted against their expected values. The average difference between observed and expected concentration varies from 1.7% to 4.3% with a maximum recorded error of 35% (Di-Acetylation, 320fMol sample). These numbers are well within the error estimates of expected peptide count per sample (**80% purity** per synthetic peptide plus errors introduced during dilutions).

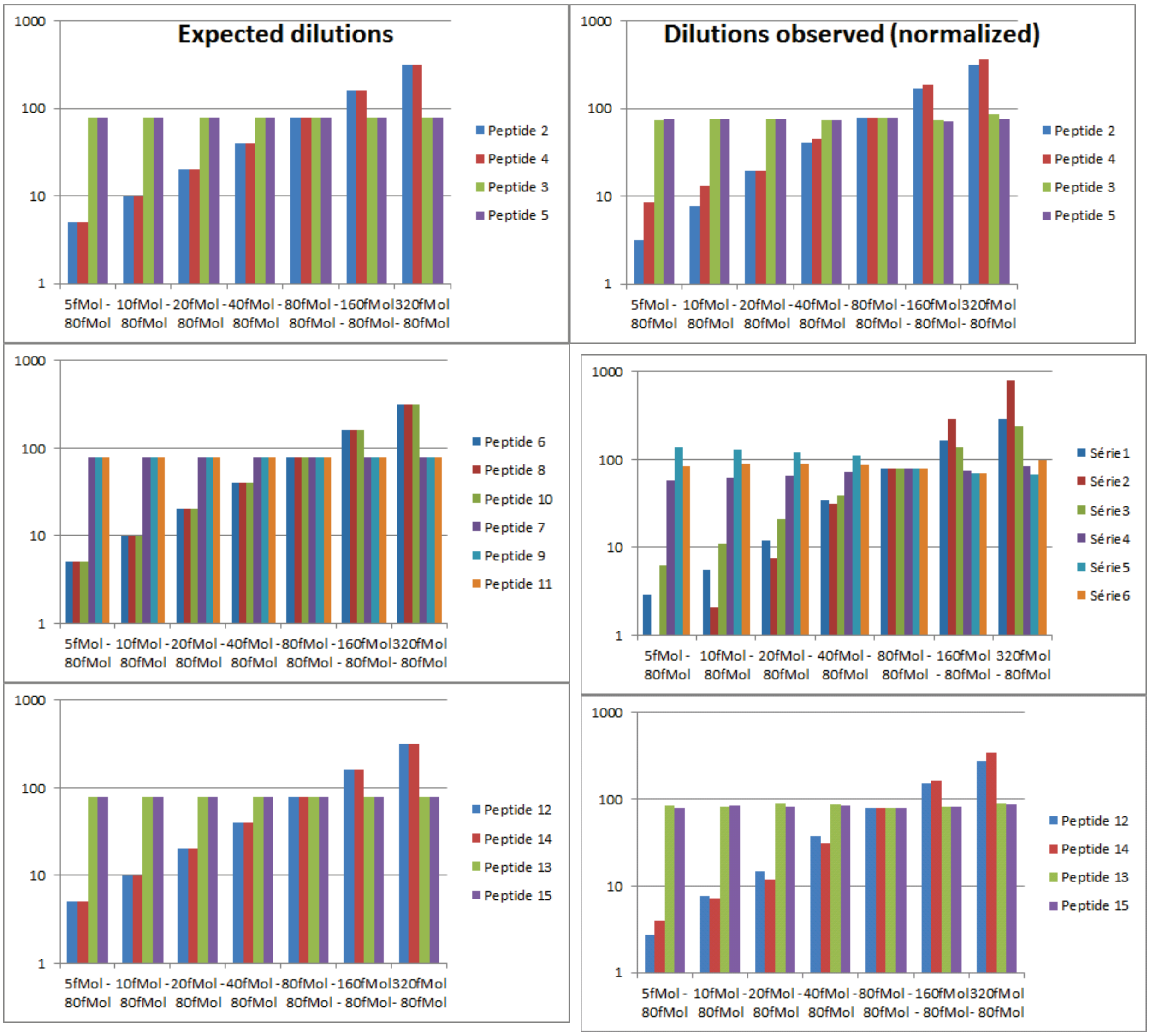
Expected dilutions
Dilutions observed (normalized)
1000
100
10
1
5fMol - 80fMol
10fMol - 80fMol
20fMol - 80fMol
40fMol - 80fMol
80fMol - 80fMol
160fMol - 80fMol
320fMol - 80fMol
Peptide 2
Peptide 4
Peptide 3
Peptide 5
Peptide 6
Peptide 8
Peptide 10
Peptide 7
Peptide 9
Peptide 11
Série1
Série2
Série3
Série4
Série5
Série6
Peptide 12
Peptide 14
Peptide 13
Peptide 15

Conclusion

Iso-PeptidAce was successfully tested for three types of peptide positional isomers on a Q-Exactive. Peptide fragmentation was done in HCD, but in theory should work equally well with other fragmentation methods. All mixed spectra samples were resolved with accuracies below the errors that were expected from sample preparation and manipulation. Furthermore, the included user interface makes the tool readily accessible to all scientists, not just bioinformaticians.

About PeptidAce

PeptidAce is an open source C# library that includes a lot of tools for peptide spectrum matching. It was based on the Morpheus open source project, with the ultimate aim of improving no-enzyme in-silico digestion of huge, unannotated transcriptome databases. It excels at identifying non tryptic peptides and can identify peptides with non-standard fragmentation patterns. It can also be used for deep spectrum annotation, matching peaks to all types of fragments and modifications. The source code and the documentation of the library are accessible from the github page:
https://github.com/olivierlizotte/PeptidAce

About Iso-PeptidAce

The user interface for Iso-PeptidAce is built using .Net 4. It is compatible with 64 bit versions of Windows 7 (and up). To work with Thermo instruments, MsFileReader (64 bit version) must be installed first. Iso-PeptidAce installer can be downloaded for free from IRIC's Proteomic Platform website:
http://proteomics.iric.ca/tools/Iso-PeptidAce

1. Kessner, D., Chambers, M., Burke, R., Agus, D. & Mallick, P. Bioinformatics 24, 2534-2536 (2008).
